# Large-scale *in-silico* statistical mutagenesis analysis sheds light on the deleteriousness landscape of the human proteome

**DOI:** 10.1038/s41598-018-34959-7

**Published:** 2018-11-19

**Authors:** Daniele Raimondi, Gabriele Orlando, Francesco Tabaro, Tom Lenaerts, Marianne Rooman, Yves Moreau, Wim F. Vranken

**Affiliations:** 1 Interuniversity Institute of Bioinformatics in Brussels, ULB-VUB, La Plaine Campus, Triomflaan, 1050 Brussels Belgium; 2 ESAT-STADIUS, KU Leuven, Kasteelpark Arenberg 10, 3001 Leuven, Belgium; 3 Structural Biology Brussels, Vrije Universiteit Brussel, Pleinlaan 2, 1050 Brussels, Belgium; 4 Machine Learning Group, ULB, La Plaine Campus, 1050 Brussels, Belgium; 5 Imec, 3001 Leuven, Belgium; 6 Department of BioModeling, BioInformatics & BioProcesses, Université Libre de Bruxelles, 1050 Brussels, Belgium; 7 Institute of Biosciences and Medical Technology, Arvo Ylpőn katu 34, 33520 Tampere, Finland

## Abstract

Next generation sequencing technologies are providing increasing amounts of sequencing data, paving the way for improvements in clinical genetics and precision medicine. The interpretation of the observed genomic variants in the light of their phenotypic effects is thus emerging as a crucial task to solve in order to advance our understanding of how exomic variants affect proteins and how the proteins’ functional changes affect human health. Since the experimental evaluation of the effects of every observed variant is unfeasible, Bioinformatics methods are being developed to address this challenge *in-silico*, by predicting the impact of millions of variants, thus providing insight into the deleteriousness landscape of entire proteomes. Here we show the feasibility of this approach by using the recently developed DEOGEN2 variant-effect predictor to perform the largest *in-silico* mutagenesis scan to date. We computed the deleteriousness score of 170 million variants over 15000 human proteins and we analysed the results, investigating how the predicted deleteriousness landscape of the proteins relates to known functionally and structurally relevant protein regions and biophysical properties. Moreover, we qualitatively validated our results by comparing them with two mutagenesis studies targeting two specific proteins, showing the consistency of DEOGEN2 predictions with respect to experimental data.

## Introduction

The next-generation sequencing revolution is providing an unprecedented amount of human sequence variation data^1^, allowing bioinformatics to address the challenging task of the *in-silico* interpretation of the phenotypic effects of genetic variants^2,3^. This presents many technical and scientific difficulties that are being constantly addressed by advances in the field^2^. The most common form of variation in our genome comprises changes of single nucleotides. When such variants occur in the exome, the protein-coding regions of our genome, they may alter the amino-acid encoded by the codon, thus causing Single Amino-acid Variants (SAVs) in the corresponding protein sequence. The exome indeed contains the vast majority of the variants identified in Mendelian disorders^4^ and many bioinformatics approaches are therefore devoted to the prediction of the effects of SAVs in these regions^5–12^.

To improve our understanding of how variants affect the function of specific proteins, the effects of SAVs can sometimes be experimentally evaluated. A common strategy is mutagenesis scanning by a selected amino acid, usually alanine but also glycine, proline or cysteine^13^ followed by biophysical measurements^14^. By identifying mutations that affect protein function, functionally relevant regions are then located^15,16^. Although very useful, the major limitation of this method is that it does not give a complete picture of which mutations to which amino acids affect protein function the most (or least): ideally, the experimental mutagenesis would involve the substitution of each residue with all other 19 amino-acids. Unfortunately, such an analysis remains experimentally unfeasible^13,17,18^, even on limited sets of proteins. On the other hand, an accurate and fast computational tool can predict the likely impact of millions of variants at an extremely low cost. Such *in-silico* mutagenesis studies have already been performed for specific proteins^13,14,17,19^, suggesting that the interpretation of these results may help i) targeting further *in-vitro* experimental verification and ii) providing actual insight into the *deleteriousness landscape* of the protein under investigation, for example by highlighting putative functionally or structurally relevant sites^17^.

Recently we developed DEOGEN2^20^, an accurate Machine Learning-based variant-effect predictor able to predict the deleterious or neutral impact of SAVs on human proteins. DEOGEN2 contextualizes SAVs from different points of view, ranging from molecular and evolutionary aspects to the involvement of the affected genes in pathways and diseases and uses a Random Forest predictor to aggregate these heterogeneous sources of information into a single deleteriousness score. In this paper, we apply this predictor to assess the impact of every possible SAV on 15000 human proteins, so investigating a total of 170 million variants. To the best of our knowledge, this is the broadest *in-silico* mutagenesis performed so far, and we analyse the resulting extensive corpus of data to investigate how the predicted deleteriousness trends relate with known functionally relevant regions in proteins, such as active sites, modified residues, interaction patches as well as structurally relevant regions such as secondary structures and domains. We show that DEOGEN2 is able to pick up these important regions as *hot-spots* for deleteriousness, despite not explicitly including any specific functional or structural annotations in its prediction. The machine learning method on which DEOGEN2 is based is therefore able to extrapolate functional information from features related to the deleteriousness of variants, such as evolutionary conservation and gene-level information. Each one of these features is related to protein function^21^, but their integration in DEOGEN2 provides a combined view on how the single evidences for the deleteriousness of a SAV impact the function of the protein, the genes and the pathways involved. For example, in^10^ we showed how, regardless of the evolutionary conservation scores, SAVs occurring on the Olfactory Signaling Pathway (REACT_15488) are extremely likely to be neutral.

In addition, we qualitatively validated these predictions in two case-studies: the Human Glucokinase protein and the Melanocortin Receptor 4 protein. In the first case, we analyzed the predicted *deleteriousness landscape* and we blind-tested DEOGEN2 on 24 Glucokinase variants that were not present in its training set, showing that our method correctly identifies them as deleterious. For the Melanocortin receptor, we blind-tested the DEOGEN2 predictions on 159 experimentally annotated variants extracted from^17^, showing that our predictor is able to distinguish between neutral SAVs and ones with functional consequences.

## Methods

### Datasets

From the July 2017 version of Swiss-prot^22^ we retrieved all the human proteins for which the existence is supported by experimental evidence at the protein level. The resulting SP17 dataset contains 15009 proteins totalling 8939795 residues. The protein sequence-level annotations for the analysis, were collected from UniprotKB^23^. In particular, we downloaded from Uniprot all the available functional annotations, post-translational modification, secondary structure, transmembrane and domain annotations (see Suppl. Material for more details). Interaction patches annotations were retrieved from Instruct^18^, which contains 11470 experimentally determined binary interactions between 3627 proteins.

### DEOGEN2 predictor

In recent work, we developed DEOGEN^10^ and DEOGEN2^20^, which predict the deleterious or neutral outcome of Single Aminoacid Variants (SAVs). In particular, DEOGEN2 has been designed with the proteome-scale screening of the effects of SAVs in mind. Its predictions are obtained with a Random Forest^24,25^ model that contextualizes each SAV with 11 features related to evolutionary, molecular, domain-level, gene-level, interaction and pathway-levels aspects of cell life^20^. DEOGEN2 is publicly available at https://deogen2.mutaframe.com. It has been extensively validated and its performance compares positively with state of the art predictors, both in cross-validation and blind test settings^20^. In this study we used DEOGEN2 to predict, for every residue *p* in every protein *P* in SP17, the outcome of all the possible 19 Single Aminoacid Substitutions (SAVs) that can occur, thus performing a full *in-silico* mutagenesis, involving a total of 169856105 mutations.

### Analysis

The p-values computed in this study are, if not otherwise specified, two-tailed Wilcoxon ranksums tests performed with the scipy^26^ library. The correlations are Pearson’s correlation coefficients computed with the same library.

## Results

### Uncovering statistical trends of deleteriousness in Human proteins

**Figure 1:**
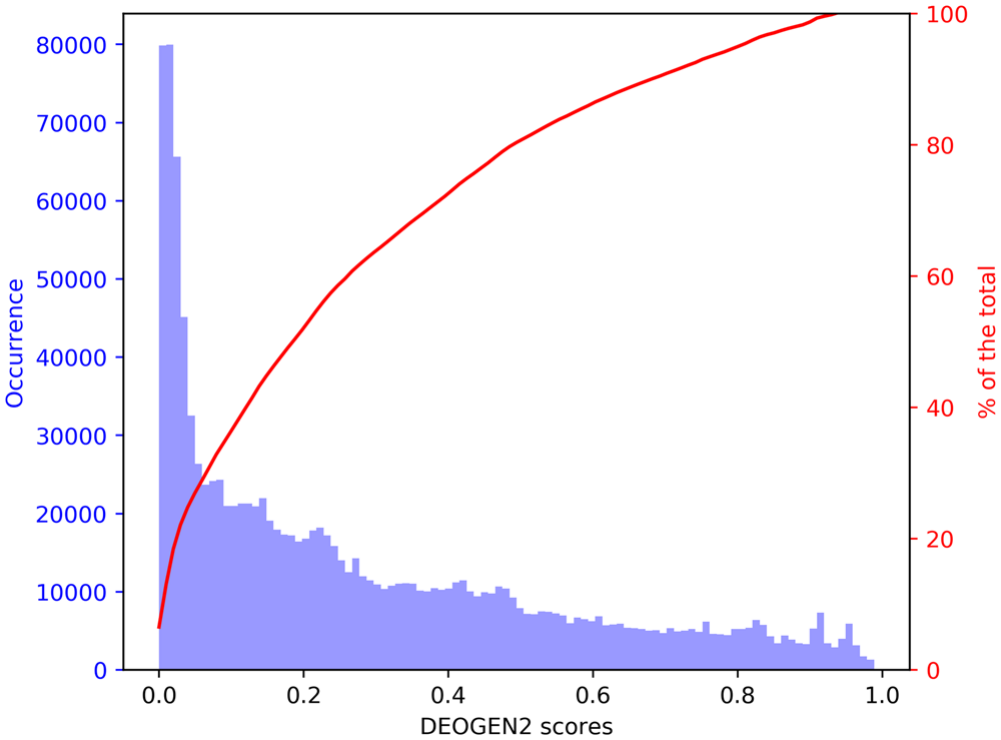
Figure showing the distribution of the 170 million DEOGEN2 predicted scores on the SP17 dataset, indicating also the cumulative distribution in red.

The SP17 dataset contains 15009 human proteins that were experimentally validated (see Methods). We selected only proteins for which high-quality annotations are available, as our goal is to investigate the relationship between deleteriousness predictions and biological and molecular aspects of the cell as annotated in UniprotKB3^27^. We used our DEOGEN2 variant-effect prediction method (see Methods) to perform a comprehensive *in-silico* mutagenesis over these proteins by predicting the outcome of every possible Single Aminoacid Variant (SAV) occurring on every sequence position. Figure 1 shows the distribution of the deleteriousness score predicted by DEOGEN2 scores for the nearly 170 million of SAVs considered in this analysis. The majority (57.6%) of these variants have a neutral or nearly-neutral predicted outcome (values < 0.3) while fewer (17.8%) SAVs yield likely deleterious predictions (values > 0.6) and even fewer (2%) have extremely high deleteriousness scores (values > 0.9). This behavior is consistent with the established hypothesis that the vast majority of the observed genetic exomic variation is neutral or nearly neutral^28,29^, even when considering all possible variants and not only typical nucleotide transitions or transversions.

**Figure 2:**
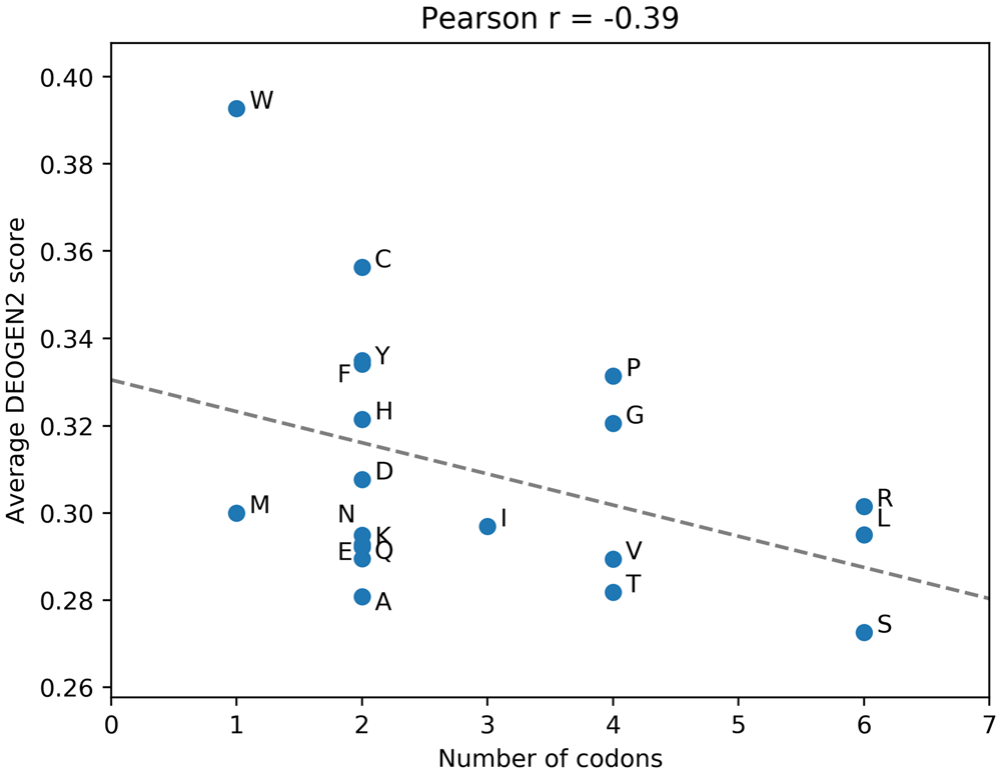
Figure showing the average DEOGEN2 scores (computed on SP17) plotted against the number of codons encoding the mutated aminoacid. Aminoacids encoded by few codons are more likely to host deleterious variants, with the exception of Met.

We first investigated general trends that can be observed in the DEOGEN2 predictions. Figure 2 shows the average deleteriousness scores for SAVs leading to the 20 possible amino-acids, sorting them in function of the number of codons encoding them (on the x axis). The DEOGEN2 mean predicted deleteriousness on the entire SP17 is negatively correlated (r = −0.39, p value = 0.08622) with the number of codons encoding a particular residue. Mutations into amino acids encoded by fewer codons therefore tend to be more deleterious, which is consistent with the observed optimality of the genetic code^30^. This suggests that when we average the effect of every possible variant *v* over all the possible regions and local sequence environments in which *v* can occur, thus levelling out the unique structural and biophysical conditions of each occurrence of v, the remaining signal that we can detect relates to the evolutionary signal that shaped the genetic code toward this particular configuration.

Met is an exception to this behavior, since it has low deleteriousness scores despite being encoded by only one codon. A likely cause is that Met corresponds to the most common start codon (AUG), leading to an over-representation of Met residues in the N-terminal of proteins, which are associated with lower deleteriousness scores (see Suppl. Figs S1–2). The observation that especially aromatic (Trp,Tyr, Phe, His) amino acids tend to lie above the regression line, while typical hydrophilic ones (Ser, Glu, Asp, Asp, Gln) lie below it, may be related to the fact that the hydrophobic core of the protein is under heavier selective pressure, while exposed regions have more room for variation. We also investigated whether there is a relation between the deleteriousness of a SAV and where in the sequence it occurs. Suppl. Fig. S1 shows the distribution of the average DEOGEN2 scores (y axis) in relation with the position in the protein on which they occur, shown as percentage of the sequence length on the x axes. There is no noticeable patterns in the central part of the proteins, which is not surprising given the averaging of the scores over a large number of proteins of different sequence length. The N and C termini of the proteins, however, are enriched for neutral variants, as shown also from the analysis on the experimentally labeled variants on the Humsavar 2016 dataset (See Suppl. Fig. S2).

#### Variants occurring on functional sites are detected to be more deleterious

**Figure 3:**
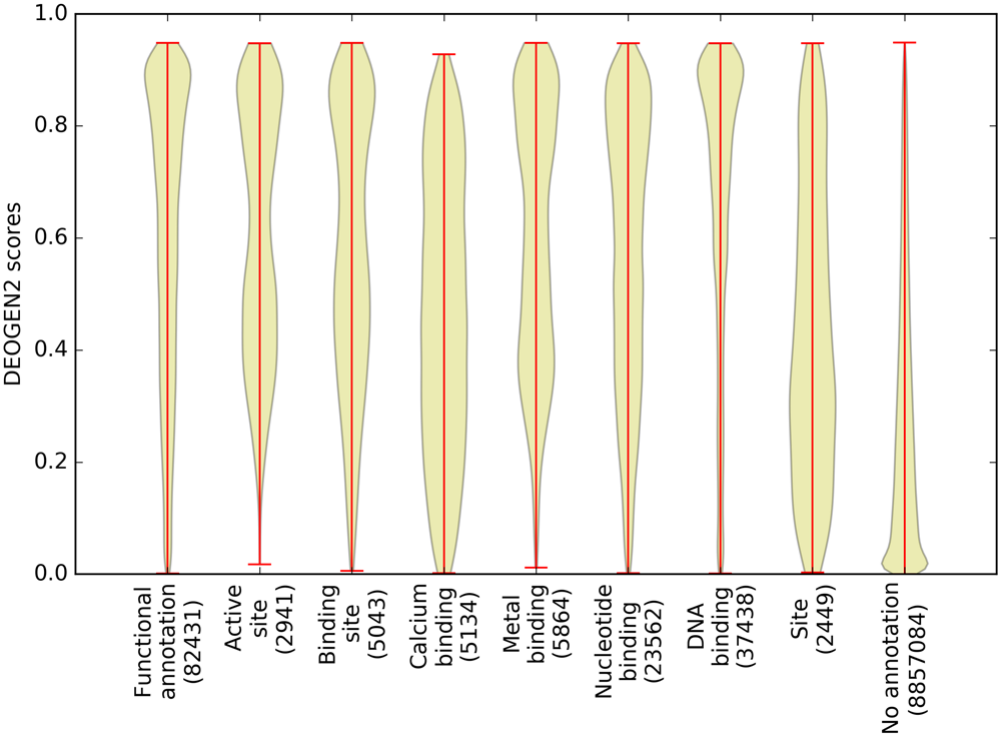
Violin plots showing the distribution of the DEOGEN2 predicted scores for regions of the protein performing different functional roles in the SP17 dataset.

The mechanisms of deleteriousness are complex and strongly influenced by the local sequence context in which the mutation occurs, effectively making each mutation *unique*, in the sense that its deleteriousness prediction needs to account for its specific position in the protein sequence, which is linked to the structure and function of the affected region^31,32^. It is well known that some regions in proteins carry out roles that can be fulfilled whilst tolerating more amino acid variation (*e.g*. linker regions, surface residues not involved in interaction patches)^33^, while other parts of the proteins respect stricter sequence constraints and preserve their function through purifying selection. To illustrate this connection, we annotated the proteins in SP17 with UniprotKB^23^ residue-level functional annotations and investigated how the distribution of the DEOGEN2 predictions to all amino acids varies between residues involved in different functional classes. Figure 3 shows these results for the 9 different classes of annotation we retrieved (see Suppl. Material for the full list), with the “Functional annotation” class grouping together all the residues comprised by these classes. “Active site” indicates that the residues are directly involved in the activity of an enzyme (e.g. proton exchange), “binding site” indicates that the residues have a role in binding a ligand (e.g a substrate or a carbohydrate). Residues or regions of the proteins able to bind with ligands such as DNA, Calcium, Metal ions and Nucleotide phosphates are annotated in the respective specific binding categories. Residues annotated as “Site” are residues that do not fall in the previous categories, for example cleavage or inhibitory sites. Finally, “No annotation” contains the vast majority of the residues without a functional annotation. The violin plots in Fig. 3 show that the DEOGEN2 predictions for residues in the “Functional annotation” class are much more deleterious than for the residues without annotation (p-value < 10^−300^). Especially DNA binding sites are highly enriched for deleterious variants, also with respect to the “Functional annotation” class (p-value < 10^−300^); Active sites, metal binding and the generic binding sites contain mostly deleterious variants, but mildly-deleterious ones, with scores between 0.4 and 0.6, are also present. Residues annotated as generic “Site” and “Calcium binding” are among the most tolerant to variation, and host both deleterious and neutral SAVs; overall, these classes are nevertheless more prone to contain deleterious variants than the unannotated residues (p-value = 10^−177^ and p-value < 10^−300^). The set of 8.86 million residues without functional annotation will contain false negatives. We indeed have to rely on the current state of knowledge while mining for Uniprot annotations, but the statistical trends are likely to be already encompassed by these data. Future studies may unveil the functional relevance of previously poorly studied residues. Suppl. Figs S3–S9 show the average deleteriousness of mutations at the residue level for each type of functional site, showing only variants occurring more than 100 times in SP17. For binding sites, all the variants in the class are generally predicted as deleterious (scores > 0.5), with some mutations between residues with similar physicochemical characteristics slightly less so (*e.g*. the positively charged Arg and Lys, and aromatic Trp, Phe and Tyr). In the case of calcium binding sites, Asp, Glu, Phe, Gly, Ile and Asn residues do not often tolerate mutations to other amino acids, which corresponds well to the typical residues found in for example EF-hand calcium binding sites^34^. Other residues like Ala, Lys, Gln, Arg, Ser, and Thr are predicted to be more tolerant to variants, except for substitutions to Trp and to a lesser extent Cys, Phe and Ile. Suppl. Fig. S5 indicates that among the residues involved in metal binding sites, almost every variant is poorly tolerated (average deleteriousness scores > 0.55) with the exception of Gly, which can be replaced with any amino acid with scores between 0.35 and 0.5. Amino acid variation in nucleotide and especially DNA binding sites is in general predicted to be highly deleterious (Suppl. Figs S6 and S7), with the exception of some conservative mutations, notably between Val and Ile. The variants with the generic “Site” annotation have the most heterogeneous substitution matrix, where mutations from Glu are generally badly tolerated, mutations from Ser are likely neutral and substitutions of residues to more unusual amino acids such as Cys and Trp are associated with deleterious predictions. Although no specific functional annotation is provided as input to DEOGEN2, from evolutionary and biophysical features our predictor can distinguish residues with enhanced functional relevance in an amino acid specific way, with often interesting asymmetric patterns appearing in the deleteriousness matrices. The relation between functional residues and evolutionary conservation is undoubtedly part of the reason for this, and this evolutionary information is indeed provided by 3 of the 11 inputs to the prediction model, with the other 8 providing protein and gene-oriented information. The distributions of the individual feature contribution to the overall DEOGEN2 prediction scores on SP17 are shown in Suppl. Fig. S10; higher scores mean that this feature has contributed more to the final prediction. For a complete description of the features see^20^ or Suppl. Material. Among the evolutionary-related features PROV is the most prominent, with also the PFAM log-odd score, the REC and ESS gene-level annotations and the pathway log-odd score relevant^20^.

#### Post-translational modified residues are hotspots for deleteriousness

Post Translational Modifications (PTMs) are another important molecular mechanism to regulate protein function, where the local sequence context is highly relevant and expected to play a role in the deleteriousness of SAVs. We retrieved all the PTM annotations available for the proteins in SP17 from UniprotKB^23^, and divided them in 4 classes: disulfide bonds, glycosylations, lipidations and “other”, which contains different types of PTMs (e.g. phosphorylation, methylation, acetylation; the full list is available in Suppl. Material.).

**Figure 4:**
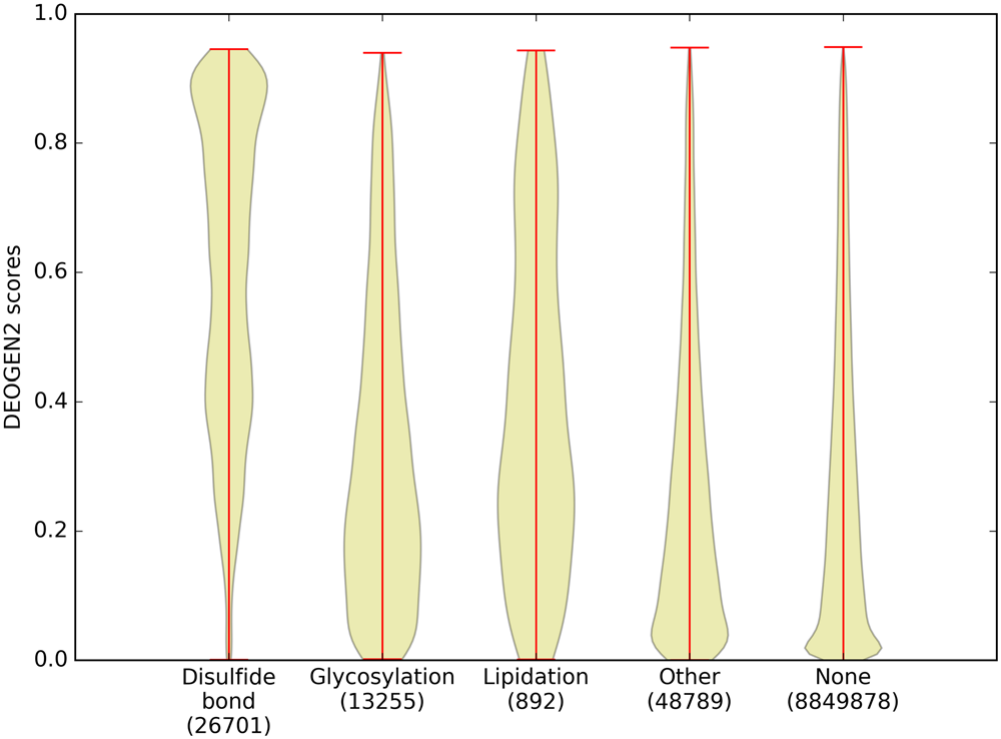
Violin plots showing the distribution of the DEOGEN2 predicted scores for residues undergoing different Post Translational Modifications in the SP17 dataset.

Disulfide bonds are strong covalent bonds occurring between the side-chains of two cysteines belonging to the same or different proteins. They are especially found in proteins in oxidative environments (typically outside the cell) and are generally structurally and functionally relevant^35^. Lipidations involve the covalent binding of a lipid to the side-chain of a residue. They can occur in many forms (e.g. palmitoylation, myristoylation or prenylation) and influence the protein function or localization^36^. Glycosylation is the process in which glycans bind the side-chain of a residue (typically oxygen linked, for example to Ser, or nitrogen linked, for example to Asn), which can influence protein folding, structure and function. Figure 4 shows the distribution of the DEOGEN2 predictions for these classes of modified residues. As previously observed, cysteines involved in disulfide bonds are highly enriched for deleterious variants, although not as strikingly as annotated in common mutation databases such as Humsavar^35^. The mutations of residues known to undergo glycosylation or lipidation are generally more likely to be deleterious than the unannotated residues in SP17 (resp. p-value = 10^−124^ and p-value = 10^−32^), also with respect to the “other” class (resp. p-value = 10^−202^ and p-value = 10^−60^), but they yield less drastically deleterious scores than oxidised cysteines (resp. p-value < 10^−300^ and p-value = 10^−103^). The mutation of residues belonging to the “other” class are significantly more deleterious than SAVs occurring on unannotated residues (p-value = 10^−89^). Suppl. Figs S11 and S12 show the average deleteriousness of variants on residues involved in glycosylations and lipidations. In the first plot, we represented N-linked and O-linked glycosylations, respectively occurring on Asn, and Thr, Ser residues. The mutation of glycosylated Asn residues is general predicted to be more deleterious than the mutations of Ser, with Thr being the most tolerant. In the lipidation case, we show the predicted outcome of the mutation of N-myristoylated glycines and of S-palmitoylated or prenylated Cys. The mutation of such Cys is scored 0.45, which is below the standard deleteriousness threshold of 0.5, indicating that the mutation of such cysteines is in general less deleterious than oxidised ones involved in disulfide bonds. The average deleteriousness score for lipidated Gly is neutral (between 0.3 and 0.4).

#### Secondary structure elements influence variants’ deleteriousness

**Figure 5:**
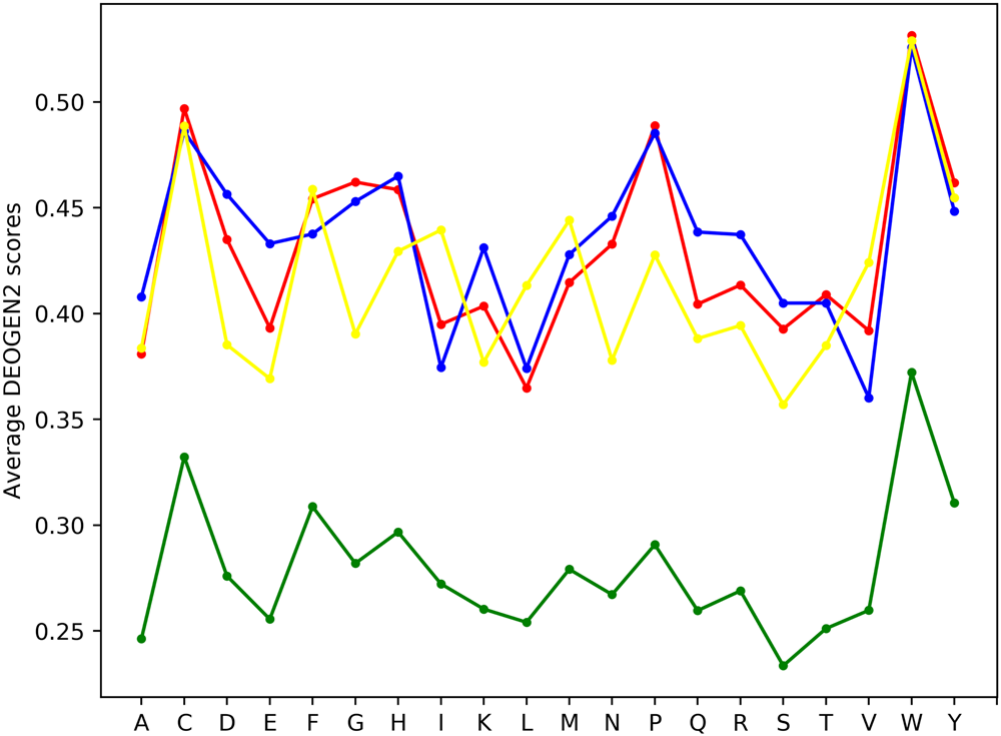
Plot showing the average DEOGEN2 predicted scores for the mutations towards the 20 aminoacids. The colored lines represent the different Secondary Structure elements, where yellow corresponds to Helix, blue to Beta-sheets, red to hydrogen bonded turns and green to unannotated structure.

The function of a protein is often intimately related with its structure, and the local sequence context has a fundamental role in determining especially the initiation of folding of particular protein regions^37^, as well as secondary structure stability^38^. To investigate the connection between the deleteriousness predictions and Secondary Structure (SS) elements, we annotated the proteins in SP17 with SS annotations extracted from UniprotKB^23^ related to the presence of Helix, Beta-sheets and hydrogen bonded Turn structures. Suppl. Fig. S13 shows the distributions of the DEOGEN2 scores mapped on these regions. SAVs in regions encompassed by these three SSs classes are more likely to be deleterious than in SS-unassigned regions (p-values < 10^−300^). This highlights that the local amino-acidic context, here as interpreted by DEOGEN2, is crucial for maintaining the interactions necessary to the form and maintain well-defined secondary structure elements. To go in more detail, we show in Fig. 5 for the mutations towards the 20 amino-acids the difference of the average DEOGEN2 predicted scores between residues in a particular SS element (helices red, beta-sheets blue and turns yellow) and SS-unassigned regions (green). In addition, Suppl. Figs S14–S16 show the average deleteriousness scores for variants occurring on residues involved in these three secondary structure elements. Even though the pattern of the residue-specific matrices is quite similar, there are clear trends in the difference between the average DEOGEN2 scores. For example, a mutation into a Gly or a Pro in helices or beta-sheets shows a marked increase in predicted deleteriousness, whereas the effect of these residues is much less pronounced in turns (yellow), which are known to often contain Gly and Pro. Mutations to charged residues such as Asp or Glu are more likely to be deleterious in beta-sheets than helices or turns^1^; mutations to rare residues with particular characteristics such as Cys and Trp always have an high chance to be deleterious. Overall beta-strands (blue) are the most sensitive to mutations, highlighting the importance of favourable amino acid interactions to stabilise these secondary structure elements. This is also in line with the observation that helices (red) are more robust to mutations than beta-strands^39^. The average deleteriousness of SAVs mapped on helical and beta-sheet regions have correlations of r = 0.5289 and r = 0.3568 with the amino-acid free energy in alpha-helix and beta-sheet conformations (Muñoz and Serrano, 1994). This may indicate that DEOGEN2 is to a certain extent able to pick up how well a residue fits within a particular Secondary Structure, without direct knowledge of secondary structure information from the input features.

**Figure 6:**
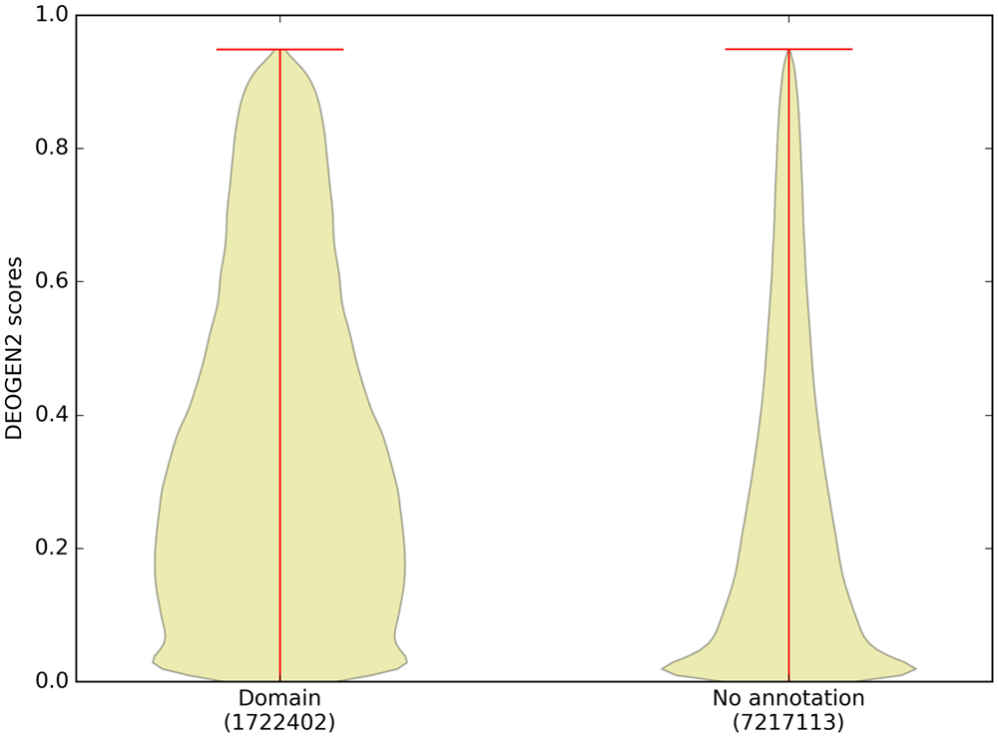
Violin plot showing the distribution of the DEOGEN2 predictions for regions of the sequences in SP17 that are mapped to a PFAM domain with respect to regions outside the known domains.

A logical further extension of this analysis are full domains, the sub-regions of proteins that are able to maintain their own independent fold. From UniprotKB we extracted domain annotations for the proteins in SP17 and in Fig. 6 we show that the DEOGEN2 predictions mapped on structured domains are more likely to host deleterious SAVs with respect to variants occurring on residues outside domains (p-value < 10^−300^). The increased deleteriousness of SAVs occurring on domains is striking but the coarse granularity of such annotations (domains can span hundreds of residues) ensures that the vast majority of the variants are still predicted as neutral, nearly neutral or mildly deleterious (scores between 0 and 0.6). One of the 11 features used in DEOGEN2 is indeed a log-odd score indicating the sensitivity of specific PFAM^40^ domains to deleterious variants, because this behavior has already been observed on dataset of clinically annotated variants^12,20^. We thus expect that variants occurring on domains that were observed in the training set are more reliably predicted, but the results in Fig. 6 show consistent results even if the domain annotations retrieved from UniprotKB comprise a combination of predicted domain boundaries from InterPro, PROSITE, Pfam and SMART^23^.

#### Interacting regions are enriched for deleterious variants

**Figure 7:**
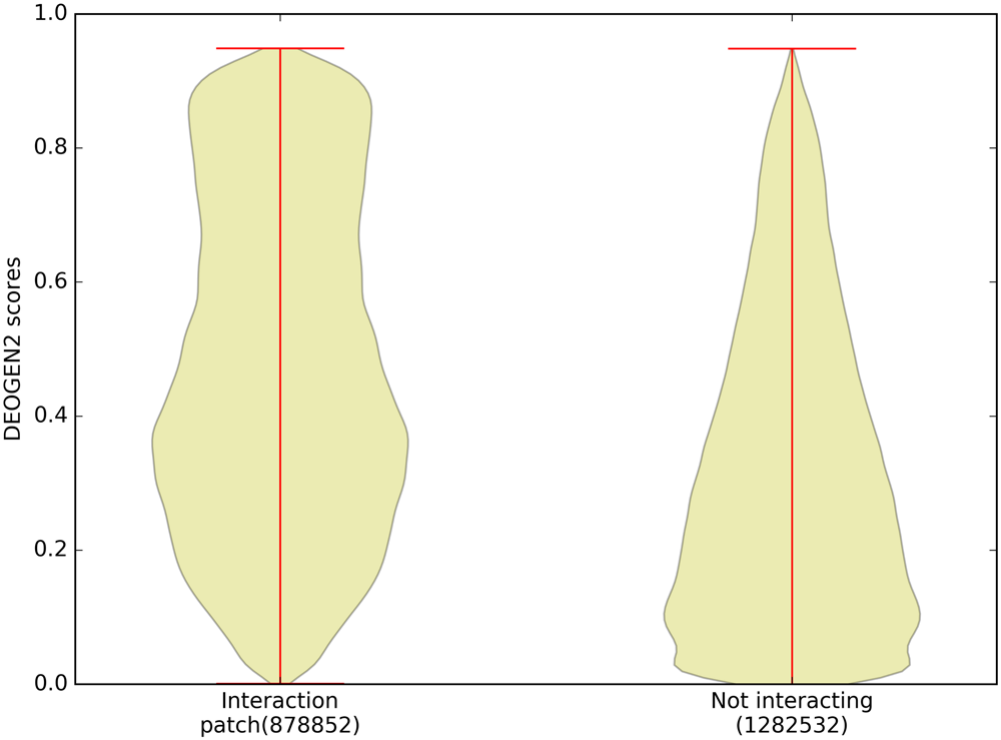
Violin plot showing the distribution of the DEOGEN2 predictions for regions of the sequences in SP17 that are mapped on Protein-Protein interaction (PPI) patches (as evinced from Instruct database^19^) with respect to regions not involved in interaction.

Proteins are *social entities* in the sense that for their functionality they need to interact with other proteins and ligands^41,42^, both by forming stable complexes or by participating in transient interactions^33^. Such interactions are in globular, folded proteins typically mediated by residues located on solvent-accessible regions of the protein. Such surface residues are generally more tolerant to deleterious variants than residues essential for the fold, which are buried in the hydrophobic core of the protein^43,44^. Residues in solvent-exposed protein-protein interaction (PPI) regions are clear exceptions to this behavior^33,45^, because they ensure favorable interactions between the binding partners. To investigate this behavior in relation to DEOGEN2 predictions, we extracted data from the Instruct^18^ database for 11470 experimentally determined binary interactions between 3627 proteins. Figure 7 shows that the DEOGEN2 predictions for SAVs located at solvent exposed PPI interaction regions tend to be more deleterious (>0.6) compared to residues in other regions of the protein (p-value < 10^−300^). A considerable amount of variants of interacting residues are still predicted as neutral, which is not unexpected as not all residues in the interacting region are equally critical for binding. Suppl. Figs S17 and S18 show, respectively, the DEOGEN2 average scores for the mutations of the residues involved in interaction patches and in any other region of the Instruct proteins. The blue colors in the second matrix correspond to the general neutrality of such variants, with respect to the ones located on the surface. For surface residues, Val to Ile and vice-versa variants are the most likely to be tolerated. Also Leu to Ile, Gln to Glu and Ser to Thr (and vice-versa) seem generally well tolerated.

#### Intra- and trans-membrane regions are more prone to host deleterious variants

**Figure 8:**
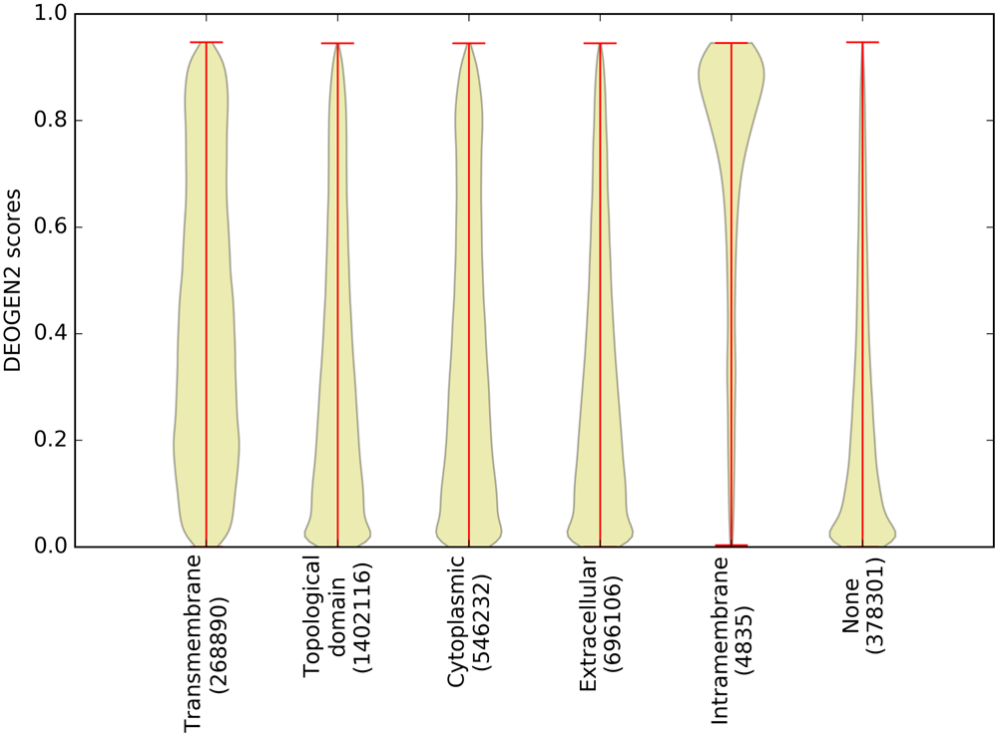
Violin plots showing the distribution of the DEOGEN2 scores for different subcellular locations spanned by transmembrane proteins in SP17.

SP17 contains 3426 proteins annotated to have at least 1 transmembrane or intra-membrane region. Membrane proteins are crucial for cell and tissue life by providing, for example, signalling functionality and response to stimuli, but they are difficult to investigate experimentally because they depend on the presence of a lipid membrane. In our *in-silico* mutagenesis, we plotted the distributions of the DEOGEN2 predicted scores for SAVs mapped on Transmembrane, Cytoplasmic, Extracellular and Intracellular regions of membrane-spanning proteins (Fig. 8). The “Topological domain” annotation indicates every subcellular localization (except the membrane itself) in which the protein chain of a membrane protein is located, and may comprise many locations (see Suppl. Material for the full list). To simplify the visualization of the results, we plotted only the two most frequent localizations, namely “Cytoplasmic” and “Extracellular”, as separate distributions. The difference between the “Transmembrane” and the “Intramembrane” annotations is that the latter applies to portions of the protein that are buried inside a membrane without fully crossing it. From Fig. 8 we see that Cytoplasmic and extracellular regions tend to contain slightly less predicted deleterious variants than transmembrane regions (p-values < 10^−300^) and definitely more tolerant to variants than intramembrane regions (p-values < 10^−300^), which is the most enriched for deleterious variants. Interestingly, in the non-membrane regions, variants of cysteine residues are predicted to be particularly deleterious in the oxidative environment of the extracellular region (see Suppl. Fig. S19) where disulfide bonds are formed, whereas in the cytoplasmic regions (see S20) the hydrophobic aromatic residues are the most sensitive. In the transmembrane regions (see Suppl. Fig. S21), mutations between typical hydrophobic amino acids (Ile, Leu, Met, Val) are completely neutral, and surprisingly only mildly sensitive to mutations to more hydrophilic amino acids. Variants of charged amino acids such as Asp and Arg, though rare in transmembrane regions, are the most sensitive in terms of deleteriousness. This might be because the definition of the limits of the transmembrane region is not always accurate: such charged residues are especially important on the cytoplasmic flank of transmembrane regions^46^. Almost any variant in the intramembrane regions (see Suppl. Fig. S22) is predicted to be deleterious, which illustrates that these regions have highly specific sequences.

### Case studies: comparison with past *in-silico* mutational screening

#### The mutational landscape of the Human Glucokinase protein

The Human Glucokinase protein (P35557) is an enzyme expressed in pancreas and liver that is involved in the phosphorylation of glucose as part of the glycogen synthesis and in the modulation of insulin secretion^47,48^. Variants on P35557 are known to cause Maturity-onset diabetes of the young 2 (MODY2)^49^ and Familial hyperinsulinemic hypoglycemia 3 (HHF3)^50^.

By performing a full *in-silico* mutagenesis of P35557 with DEOGEN2 we obtain its complete *deleteriousness landscape*, as shown in Suppl. Fig. S23, where red indicates deleterious predictions and blue indicates neutral predictions: most of the SAVs in this protein are predicted to be deleterious. An interactive landscape can also be obtained from the DEOGEN2 web server^20^ (http://deogen2.mutaframe.com). From Uniprot we then retrieved 67 experimentally determined variants and analyzed the DEOGEN2 predictions for the subset of 24 variants that are not present in its training dataset (the list is available in Suppl. Material). All of the variants in this blind set are associated with either HHF3 or MODY2 and correspond to DEOGEN2 scores greater than 0.59, suggesting their deleteriousness. Many of these variants (e.g. Ile225Met, Glu256Ala, Glu248Lys, Ser441Trp) affect the enzymatic activity, either by decreasing or increasing P35557 affinity for glucose, which may cause altered thresholds for insulin release^51,52^.

#### Functionally important residues on the Human Melanocortin receptor

**Figure 9:**
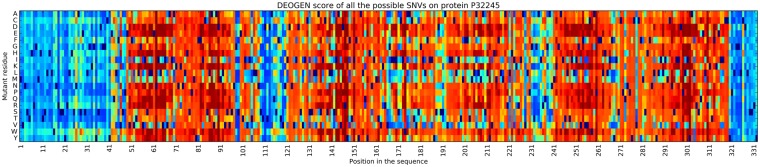
Heatmap showing the complete *deleteriousness landscape* of the Human Melanocortin receptor 4 protein. Blue-ish scores indicate neutral variants, red-ish scores indicate deleterious predictions. The blue cells correspond to the wild-type aminoacid.

The Melanocortin receptor 4 (P32245) belongs to the GPCR family of transmembrane proteins and is important for energy homeostatis and somatic growth^53^. Variants of this protein have been associated with obesity^54^ (OMIM:601665), and functional effects have been studied with *in-silico* methods^17^, where the predictions of SNAP (REFsnap) were compared with 159 SAVs for which experimental annotations are available^17^. Here we used DEOGEN2 to compute the full *in-silico* mutagenesis of P32245 (see Fig. 9) and we compared the predicted deleteriousness landscape with SNAP mutagenesis results and the experimentally validated variants.

From Fig. 9 we can see that the extracellular N-terminal residues of the protein (1–43) are generally predicted as tolerant to SAVs by DEOGEN2, with the exception of the glycosylated Asn26. The residues that are predicted to be tolerated at this position are Ser and Thr, which can also be glycosylated, and Pro, which is often found around glycosylation sites^55^. The first two transmembrane helical regions (res. 44–69 and 82–106) and the first cytoplasmic region (res. 70–81) are predicted to be generally intolerant to variation, and the first tolerant region encountered is located near the second extracellular region (res. 107–121). The fourth transmembrane helix could host some neutral variants (Val, Leu, Met, Ala), and surprisingly also towards polar residues such as Ser and Thr. This behavior is maintained until the cytoplasmic region after the fifth transmembrane helix. The C-terminal is predicted to host mostly neutral variants.

**Figure 10:**
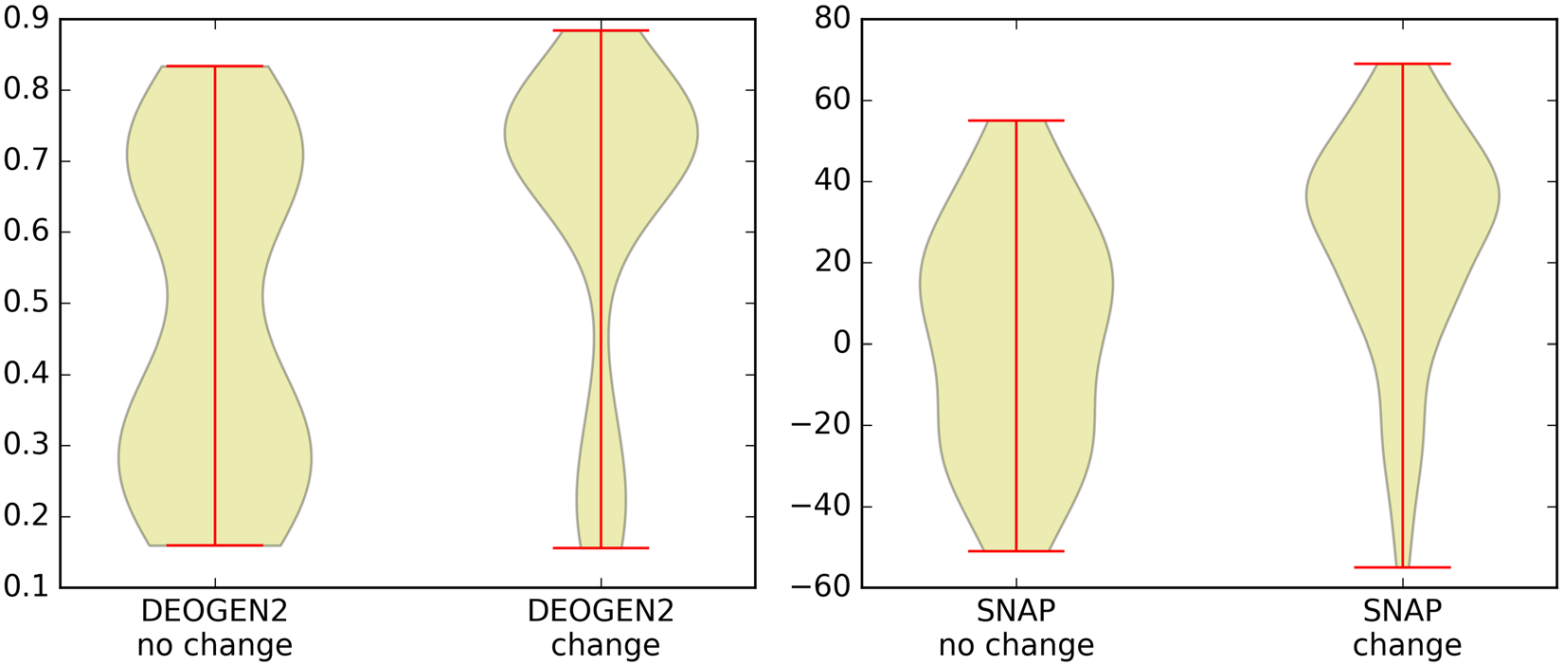
Violin plots showing the DEOGEN2 and SNAP predicted scores for the 159 experimentally annotated variants on the Melanocortin receptor 4 used as blind-test set. These variants are annotated to cause or not a functional change in the protein.

We blind-tested DEOGEN2 predictions with the 159 experimentally validated SAVs extracted from^17^, which are annotated on whether they impact the function of P32245 (change) or not (no change). These SAVs are not present in DEOGEN2 training set, and in Fig. 10 we show the distributions of the DEOGEN2 scores (left panel) and the SNAP scores (right panel) for these two classes. Both predictors discriminate between the SAVs affecting the function (DEOGEN2 p-value = 1.7 × 10^−4^, SNAP p-value = 4.15 × 10^−5^), even though DEOGEN2 is trained to identify variants that have deleterious effects on the *individual phenotype* and these annotations relate with functional changes in the protein^17^. We also investigated the level of agreement between these full mutagenesis performed by SNAP and DEOGEN2 (see Suppl. Fig. S24), obtaining a correlation of r = 0.77594, indicating even if DEOGEN2 is not specifically trained for the task of predicting the functional effect of variants, it can, to a certain extent, perform this duty.

## Discussion

In this study we performed the largest *in-silico* mutagenesis analysis to date that we are aware of, analysing nearly 170 million SAV predictions computed with our DEOGEN2 method. We chose to focus on our in-house DEOGEN2 predictor because it provides state of the art performances and is one of the most reliable tools available at the moment, which we demonstrated by different validations^20^. Another important reason is that it uses limited number of features that have been chosen because of their biological relevance for the deleteriousness prediction problem. These characteristics, coupled with the interpretable nature of Random Forest^56^, enables interpretation of the predictions, unlike, for example, meta-predictors that use the predictions of many other deleteriousness prediction methods as input.

We show that these predictions have a statistical relation with biophysical and structural aspects of proteins, for example functional sites, post-translationally modified residues and secondary structure elements, which are generally enriched with deleterious variants. The machine learning method was not provided directly with this kind of information, but is still able to infer such important sites in proteins from the generic features that were provided as input, such as evolutionary conservation and gene-level features. Moreover, we show that in functionally relevant positions the predicted deleteriousness is modulated by the type of the mutant residue, meaning that not only the average evolutionary conservation of the particular position has a relevant role in the decision, but also the types of the amino acids involved. From an analysis of the feature contributions in this mutagenesis study (see Suppl. Fig. S10), it appears that, as expected, evolutionary variant-level features are important for the final predictions, but domain-level, gene-level and pathway-level information are also crucial to provide a full characterization of the target variants and their possible biological effects on the human organism.

Whether a particular variant is deleterious or neutral is, ultimately, due to the underlying molecular changes instigated by the difference in amino acid at a specific position in the protein. It is therefore maybe not surprising that deleteriousness predictions detect positions that are known to be important for protein function and behavior. However, the reasons at the molecular level, causing a change in molecular phenotype, remain very difficult to determine, despite commendable efforts like MutPred2^57^ that try to articulate those reasons by pinpointing possible causes. Still, proteins function in complex manners, and many of the aspects we examine here are likely interconnected, for example it is well known that membrane-spanning regions form secondary structure elements with hydrogen bonds between polar atoms, but on the other hand phosphorylation sites tend to occur in loop regions of proteins^58^. Also, a particular amino acid variant that disrupts, for example, a helix and is deleterious in one protein could have a similar molecular effect in another protein but be fully neutral due to gene and pathway-level redundancies or other effects. There is therefore a substantial amount of work ahead to determine the real reasons of deleteriousness, which originate at the molecular level but propagate (or not) because of the role the protein has within the complex network of the cell. Large-scale analyses like ours that start to interconnect all these levels of complexity are likely the only way in which we can start to comprehend the real causes behind deleterious variants. They will become increasingly relevant as current shortcomings in predictions and annotations are resolved. Although the performance of DEOGEN2 is very good, and means that predicted differences between deleterious and neutral SAVs are relevant at the statistical level, this might not be the case for individual SAVs, where experimental validation remains essential. Furthermore, the quality of annotations at the residues, and especially the absence thereof, means that our observations could in reality be more pronounced, or alternatively could turn out to be invalid because of bias in the current data. Given the scale of this study, its focus on statistical differences, and the connection to known rationale about SAVs and their effect on molecular phenotype, we do think that most of our observations are likely to remain valid, and that they indicate evolutionary tendencies that are difficult to extract from individual protein families.

DEOGEN2 predictions can be obtained from the webserver http://deogen2.mutaframe.com, which provides both an interactive visualization of different aspects of the variants and the text-format predictions.

## Electronic supplementary material

Supplementary material

## Data Availability

All the data presented in the manuscript are available upon request or at http://deogen2.mutaframe.com/.

## References

[1] Lek M (2016). T. Analysis of protein-coding genetic variation in 60,706 humans. Nature.

[2] Pabinger S (2014). A survey of tools for variant analysis of next-generation genome sequencing data. Briefings in bioinformatics.

[3] Van Dijk EL, Auger H, Jaszczyszyn Y, Thermes C (2014). Ten years of next-generation sequencing technology. Trends in genetics.

[4] Rabbani B, Tekin M, Mahdieh N (2014). The promise of whole-exome sequencing in medical genetics. Journal of human genetics.

[5] Adzhubei IA (2010). A method and server for predicting damaging missense mutations. Nature methods.

[6] Calabrese R, Capriotti E, Fariselli P, Martelli PL, Casadio R (2009). Functional annotations improve the predictive score of human disease-related mutations in proteins. Hum. Mutat..

[7] Choi Y, Sims GE, Murphy S, Miller JR, Chan AP (2012). Predicting the functional effect of amino acid substitutions and indels. PloS one,.

[8] Dong C (2014). Comparison and integration of deleteriousness prediction methods for nonsynonymous SNVs in whole exome sequencing studies. Human molecular genetics.

[9] Ng PC, Henikoff S (2003). SIFT: Predicting amino acid changes that affect protein function. Nucleic Acids Res..

[10] Raimondi D (2016). Multilevel biological characterization of exomic variants at the protein level significantly improves the identification of their deleterious effects. Bioinformatics.

[11] Schwarz JM, Rodelsperger C, Schuelke M, Seelow D (2010). MutationTaster evaluates disease-causing potential of sequence alterations. Nat. Methods.

[12] Shihab HA (2013). Predicting the functional, molecular, and phenotypic consequences of amino acid substitutions using hidden Markov models. Human mutation.

[13] Bromberg Y, Rost B (2008). Comprehensive in silico mutagenesis highlights functionally important residues in proteins. Bioinformatics.

[14] Hecht M, Bromberg Y, Rost B (2013). News from the protein mutability landscape. Journal of Molecular Biology.

[15] Gårdsvoll H (2006). Characterization of the Functional Epitope on the Urokinase Receptor. Complete alanine scanning mutagenesis supplemented by chemical crosslinking. Journal of Biological Chemistry.

[16] Qin L, Cai S, Zhu Y, Inouye M (2003). Cysteine-scanning analysis of the dimerization domain of EnvZ, an osmosensing histidine kinase. Journal of bacteriology.

[17] Bromberg Y, Overton J, Vaisse C, Leibel RL, Rost B (2009). In silico mutagenesis: a case study of the melanocortin 4 receptor. The FASEB Journal.

[18] Meyer MJ, Das J, Wang X, Yu H (2013). INstruct: a database of high-quality 3D structurally resolved protein interactome networks. Bioinformatics.

[19] Saunders CT, Baker D (2002). Evaluation of structural and evolutionary contributions to deleterious mutation prediction. Journal of molecular biology.

[20] Raimondi D (2017). DEOGEN2: prediction and interactive visualization of single amino acid variant deleteriousness in human proteins. Nucleic acids research.

[21] Lee D, Redfern O, Orengo C (2007). Predicting protein function from sequence and structure. Nature Reviews Molecular Cell Biology.

[22] UniProt Consortium. UniProt: a hub for protein information. *Nucleic acids research*, **43**(D1), D204–D212 (2014). 10.1093/nar/gku989 PMC4384041 25348405

[23] Magrane, M. & UniProt Consortium. UniProt Knowledgebase: a hub of integrated protein data. *Database*, p.bar009 (2011). 10.1093/database/bar009 PMC3070428 21447597

[24] Breiman L (2001). Random forests. Mach. Learn..

[25] Pedregosa F (2011). Scikit-learn: Machine learning in Python. Journal of machine learning research.

[26] Oliphant Travis E. (2007). Python for Scientific Computing. Computing in Science & Engineering.

[27] Bamshad MJ (2011). Exome sequencing as a tool for Mendelian disease gene discovery. Nature Reviews Genetics.

[28] Kimura M (1968). Evolutionary rate at the molecular level. Nature.

[29] Ohta T (2002). Near-neutrality in evolution of genes and gene regulation. Proceedings of the National Academy of Sciences.

[30] Freeland SJ, Knight RD, Landweber LF, Hurst LD (2000). Early fixation of an optimal genetic code. Molecular Biology and Evolution.

[31] Loeb DD (1989). Complete mutagenesis of the HIV-1 protease. Nature.

[32] Markiewicz P, Kleina L, Cruz C, Ehret S, Miller C (1993). Analysis of 4000 altered Escherichia coli lac repressors resulting from suppression of nonsense mutations at 328 positions in the lacI gene. J Mol Biol.

[33] David A, Razali R, Wass MN, Sternberg MJ (2012). Protein–protein interaction sites are hot spots for disease‐associated nonsynonymous SNPs. Human mutation.

[34] Grabarek Z (2006). Structural basis for diversity of the EF-hand calcium-binding proteins. Journal of molecular biology.

[35] Raimondi D, Orlando G, Messens J, Vranken WF (2017). Investigating the Molecular Mechanisms Behind Uncharacterized Cysteine Losses from Prediction of Their Oxidation State. Human mutation.

[36] Hentschel A, Zahedi RP, Ahrends R (2016). Protein lipid modifications—More than just a greasy ballast. Proteomics.

[37] Englander SW, Mayne L (2014). The nature of protein folding pathways. Proceedings of the National Academy of Sciences.

[38] Rooman MJ, Rodriguez J, Wodak SJ (1990). Relations between protein sequence and structure and their significance. Journal of molecular biology.

[39] Abrusán G, Marsh JA (2016). Alpha helices are more robust to mutations than beta strands. PLoS computational biology.

[40] Finn RD (2016). The Pfam protein families database: towards a more sustainable future. Nucleic Acids Res..

[41] Goh KI (2007). The human disease network. Proceedings of the National Academy of Sciences.

[42] Rolland T (2014). A proteome-scale map of the human interactome network. Cell.

[43] Wang Z, Moult J (2001). SNPs, protein structure, and disease. Human mutation.

[44] Yue P, Moult J (2006). Identification and analysis of deleterious human SNPs. Journal of molecular biology.

[45] Bogan AA, Thorn KS (1998). Anatomy of hot spots in protein interfaces. J Mol Biol.

[46] Baker JA, Wong WC, Eisenhaber B, Warwicker J, Eisenhaber F (2017). Charged residues next to transmembrane regions revisited:“Positive-inside rule” is complemented by the “negative inside depletion/outside enrichment rule”. BMC biology.

[47] Iynedjian PB (2009). Molecular physiology of mammalian glucokinase. Cellular and Molecular Life Sciences.

[48] Kawai S, Mukai T, Mori S, Mikami B, Murata K (2005). Hypothesis: structures, evolution, and ancestor of glucose kinases in the hexokinase family. Journal of bioscience and bioengineering.

[49] Stoffel M (1992). Human glucokinase gene: isolation, characterization, and identification of two missense mutations linked to early-onset non-insulin-dependent (type 2) diabetes mellitus. Proceedings of the National Academy of Sciences.

[50] Glaser B (1998). Familial hyperinsulinism caused by an activating glucokinase mutation. New England Journal of Medicine.

[51] Beer NL (2012). Insights into the pathogenicity of rare missense GCK variants from the identification and functional characterization of compound heterozygous and double mutations inherited in cis. Diabetes care.

[52] Gidh-Jain M (1993). Glucokinase mutations associated with non-insulin-dependent (type 2) diabetes mellitus have decreased enzymatic activity: implications for structure/function relationships. Proceedings of the National Academy of Sciences.

[53] Farooqi IS (2003). Clinical spectrum of obesity and mutations in the melanocortin 4 receptor gene. New England Journal of Medicine.

[54] Hinney A (1999). Several mutations in the melanocortin-4 receptor gene including a nonsense and a frameshift mutation associated with dominantly inherited obesity in humans. The Journal of Clinical Endocrinology & Metabolism.

[55] Christlet THT, Veluraja K (2001). Database analysis of O-glycosylation sites in proteins. Biophysical journal.

[56] Gazzo A (2017). Understanding mutational effects in digenic diseases. Nucleic acids research.

[57] Pejaver, V. *et al*. MutPred2: inferring the molecular and phenotypic impact of amino acid variants. *BioRxiv*, 134981 (2017). 10.1038/s41467-020-19669-x PMC7680112 33219223

[58] Zhao YW, Lai HY, Tang H, Chen W, Lin H (2016). Prediction of phosphothreonine sites in human proteins by fusing different features. Scientific reports.

