# Supplementary material for: Large-scale in-silico statistical mutagenesis analysis sheds light on the deleteriousness landscape of the human proteome
Source: Sci Rep. 2018 Nov 19;8:16980. doi: 10.1038/s41598-018-34959-7 (PMC6242909; doi:10.1038/s41598-018-34959-7)
Supplement: Supplementary file 1 — Supplementary material [file 41598_2018_34959_MOESM1_ESM.pdf]

Large-scale *in-silico* statistical mutagenesis analysis sheds light on the deleteriousness landscape of the human proteome.

(Supplementary Material)

Daniele Raimondi, Gabriele Orlando, Francesco Tabaro, Tom Lenaerts, Marianne Rooman, Yves Moreau, Wim F. Vranken

Uniprot annotations

The full list of annotations describing regions of interest in the protein sequence that are available on Uniprot can be found at http://www.uniprot.org/help/sequence_annotation . To annotate functional sites we used the following four classes (reported from the Uniprot website with the corresponding link to the specific pages):

Active site	Amino acid(s) directly involved in the activity of an enzyme
Metal binding	Binding site for a metal ion
Binding site	Binding site for any chemical group (co-enzyme, prosthetic group, etc.)
Site	Any interesting single amino acid site on the sequence

To annotate Post-translational modifications we used the following classes:

Modified residue	Modified residues excluding lipids, glycans and protein cross-links
Lipidation	Covalently attached lipid group(s)
Glycosylation	Covalently attached glycan group(s)
Disulfide bond	Cysteine residues participating in disulfide bonds
Calcium binding	Position(s) of calcium binding region(s) within the protein
Nucleotide binding	Nucleotide phosphate binding region (ATP-binding, cAMP-binding, cGMP-binding, FAD, FMN, GTP-binding, NAD, NADP.)

The secondary structure annotations are the following:

Helix	Helical regions within the experimentally determined protein structure
Turn	Turns within the experimentally determined protein structure
Beta strand	Beta strand regions within the experimentally determined protein structure

The annotations related to transmembrane proteins are the following:

Topological domain	Location of non-membrane regions of membrane-spanning proteins
Transmembrane	Extent of a membrane-spanning region
Intramembrane	Extent of a region located in a membrane without crossing it

The annotations related to domain are extracted from:

Domain	Position and type of each modular protein domain

DEOGEN2 features description

Feature	Reference	Name
PROVEAN score	Choi et al, *PloS One* 2012	PROV
Conservation Index (12,13)	Calabrese et al., *Bioinformatics* 2009	CI
Mutant/wildtype log-odd ratio (12)	Raimondi et al., *Bioinformatics* 2016	LOR
Early Folding predictions	Raimondi et al., *SciRep* 2017	EF
PFAM log-odd score (17)	Shihab et al., 2013	PF
Interaction patches annotation (19)	Meyer et al., *Bioinformatics* 2013	INT
RVIS (20)	Petrovski et al., *PloS Gen.* 2013	RVIS
GDI (21)	Itan et al., *PNAS* 2015	GDI
Recessiveness index (22)	MacArthur et al., *Science* 2012	REC
Gene essentiality (23)	Georgi et al., *Plos Gen.* 2013	ESS
Pathway log-odd score (12,13)	Calabrese et al., *Bioinformatics* 2009	PATH

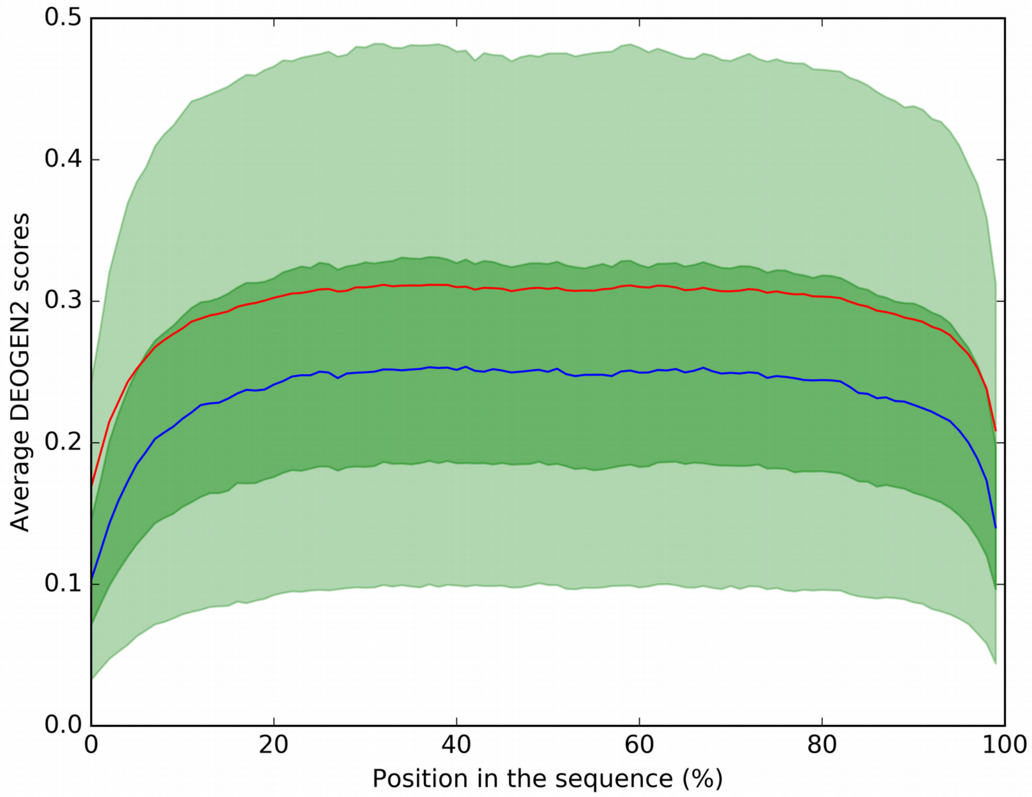

Supplementary Figures

Fig. S1: Distributions of the DEOGEN2 scores, averaged for each position in the sequence for every protein in SP17. The sequence position is represented as percentage of the protein length. The red line indicates the mean of the distribution and the blue line the median. The dark green region indicates the 40-60 quartiles region and the light green one corresponds to the 25-75 quartiles.

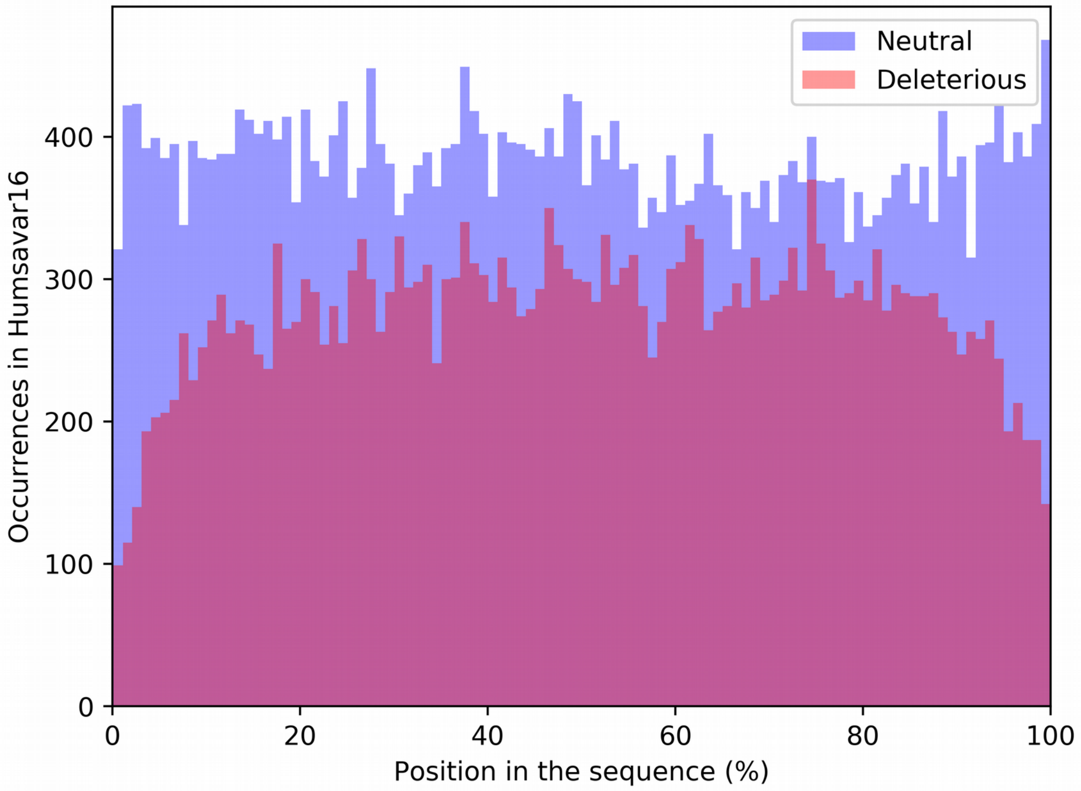

Fig. S2: Plot showing the distribution of the deleterious and neutral Humsavar 2016 annotations with respect to their position in the protein sequence.

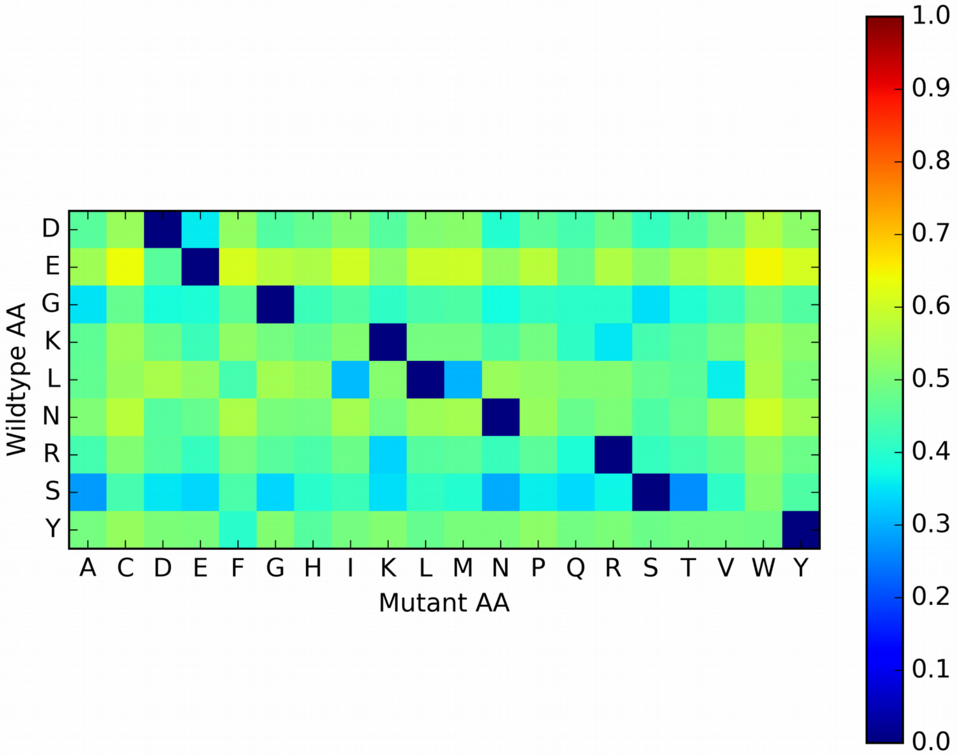

Fig. S3: Matrix showing the average deleteriousness prediction scores for variants mutating a wildtype residue (Y axis) into one of the possible 20 amino acids (X axis) when the wildtype residue is involved in a binding site. (see Suppl. Mat. Uniprot Annotations details)

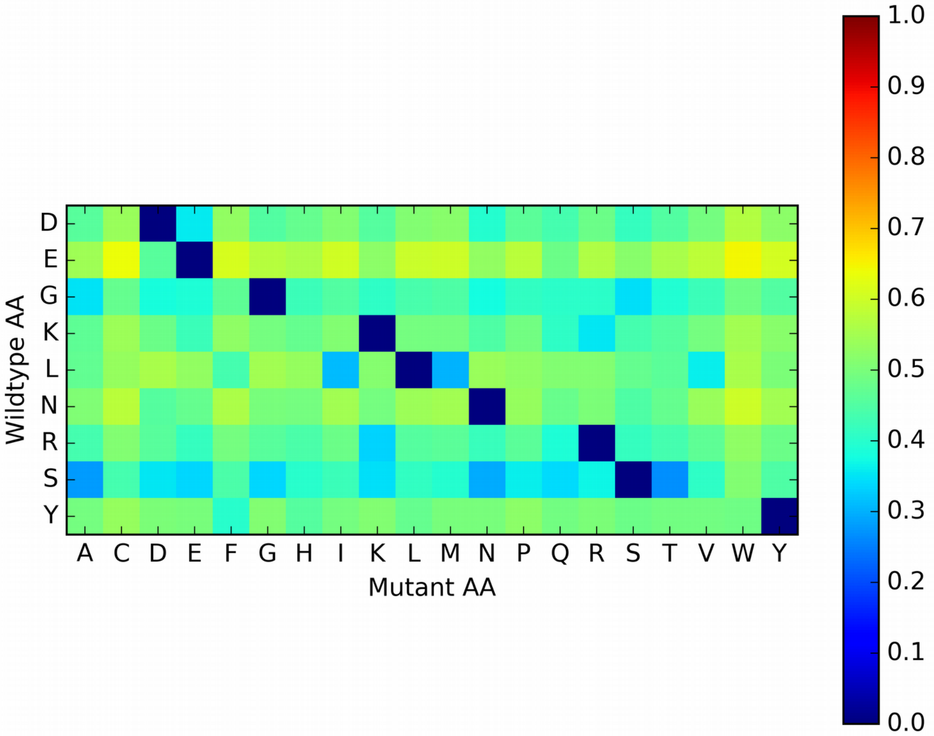

Fig. S4: Matrix showing the average deleteriousness prediction scores for variants mutating a wildtype residue (Y axis) into one of the possible 20 amino acids (X axis) when the wildtype residue is involved in a generic “site” annotation from Uniprot (see Suppl. Mat. Uniprot Annotations details).

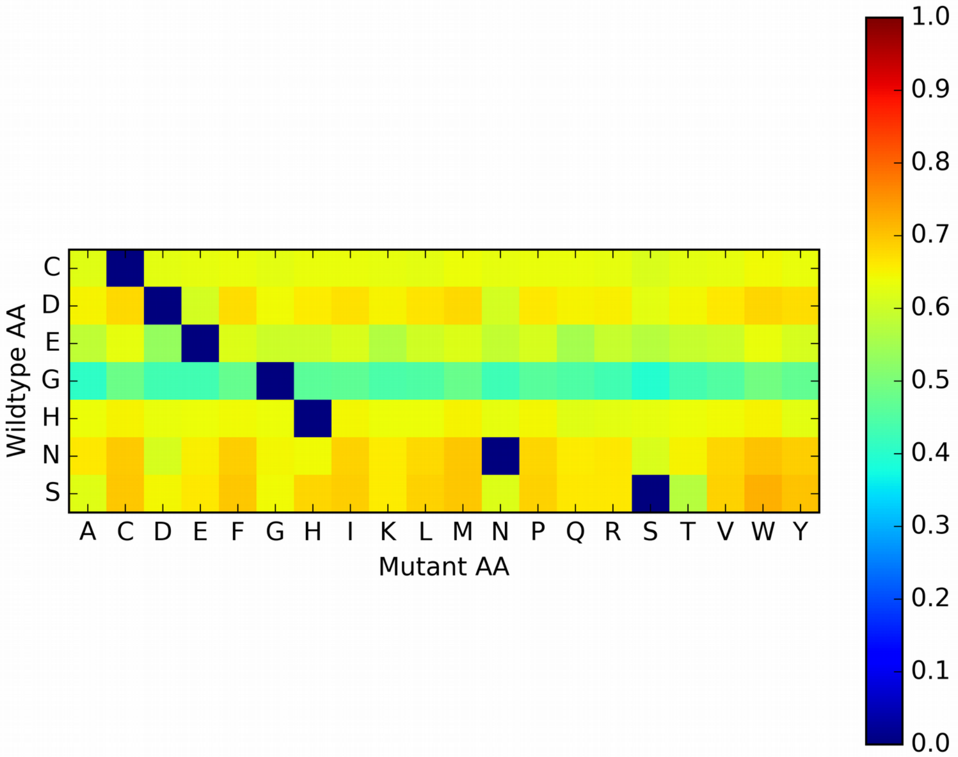

Fig. S5: Matrix showing the average deleteriousness prediction scores for variants mutating a wildtype residue (Y axis) into one of the possible 20 amino acids (X axis) when the wildtype residue is involved in a metal binding site (see Suppl. Mat. Uniprot Annotations details).

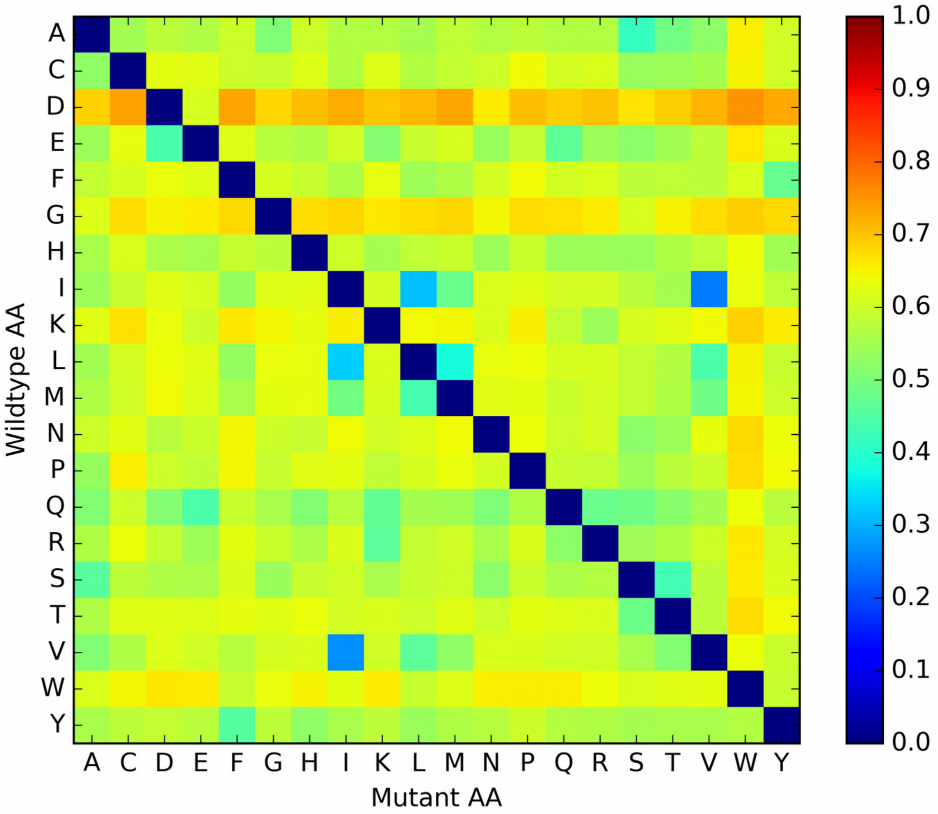

Fig. S6: Matrix showing the average deleteriousness prediction scores for variants mutating a wildtype residue (Y axis) into one of the possible 20 amino acids (X axis) when the wildtype residue is involved in a nucleotide binding site (see Suppl. Mat. Uniprot Annotations details).

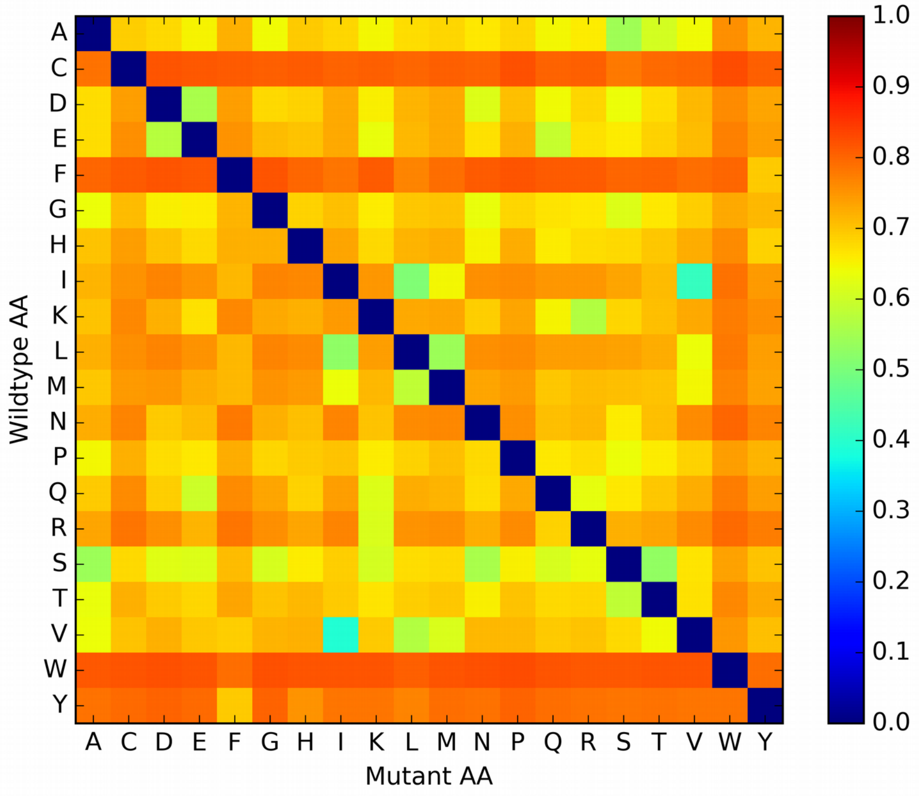

Fig. S7: Matrix showing the average deleteriousness prediction scores for variants mutating a wildtype residue (Y axis) into one of the possible 20 amino acids (X axis) when the wildtype residue is involved in a DNA binding site (see Suppl. Mat. Uniprot Annotations details).

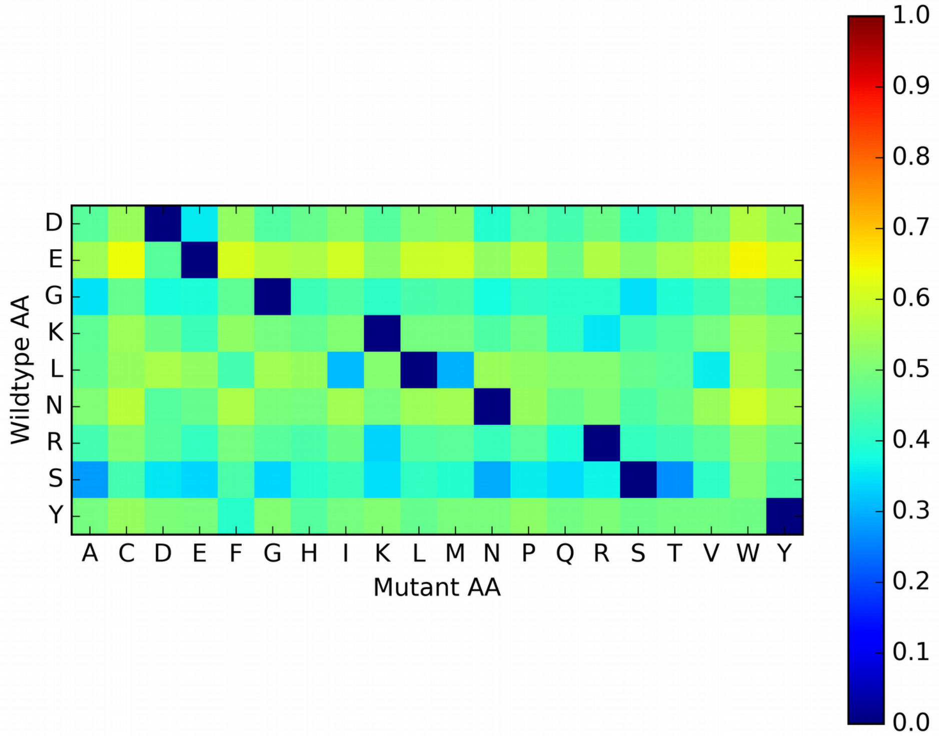

Fig. S8: Matrix showing the average deleteriousness prediction scores for variants mutating a wildtype residue (Y axis) into one of the possible 20 amino acids (X axis) when the wildtype residue is involved in an acrive site (see Suppl. Mat. Uniprot Annotations details).

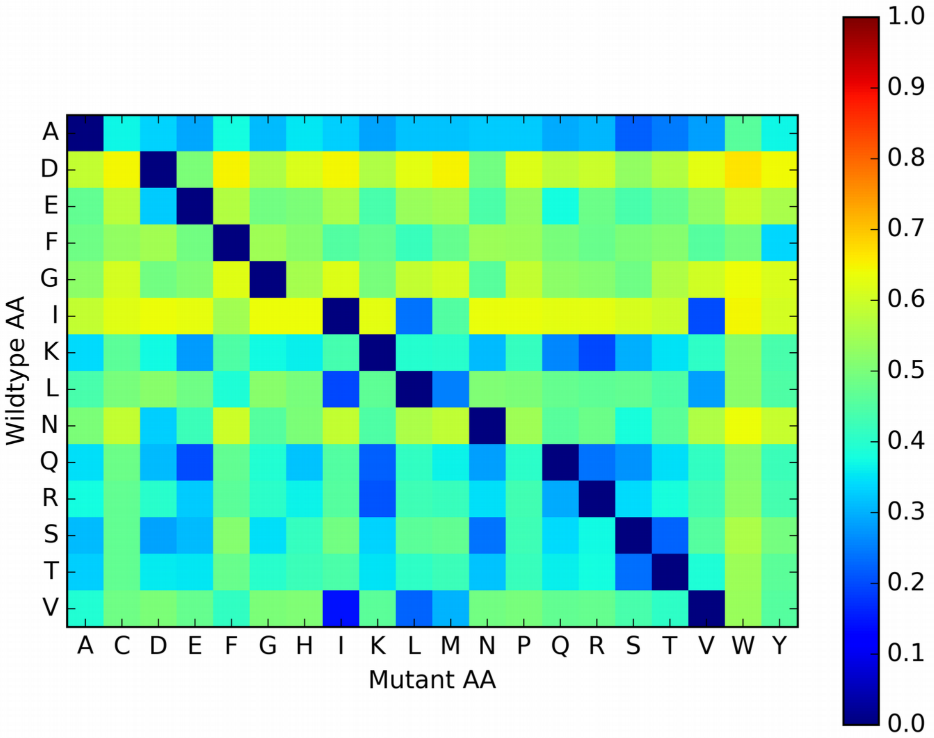

Fig. S9: Matrix showing the average deleteriousness prediction scores for variants mutating a wildtype residue (Y axis) into one of the possible 20 amino acids (X axis) when the wildtype residue is involved in a DNA binding site (see Suppl. Mat. Uniprot Annotations details).

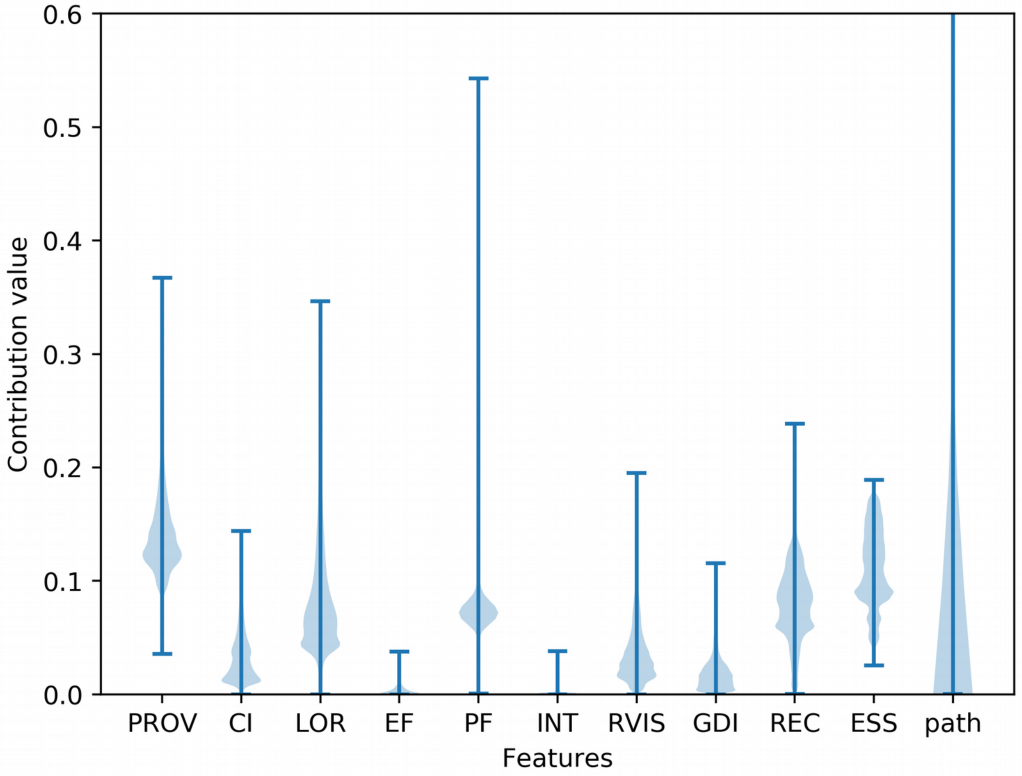

Fig. S10: Violin plot showing the contributions of the features used within DEOGEN2. PROV, CI and LOR are evolutionary-related features computed from Multiple Sequence Alignments. EF relates with the probability for the target residue to be involved in the earliest stages of protein folding. PF is the PFAM-domain likelihood of hosting deleterious variants, INT indicates if the variant occurs on an interaction patch. RVIS, GDI, REC and ESS contextualize the relevance of the gene for the organism from different points of view. PATH indicates the likelihood of the affected pathways to be involved in diseases. A full explanation can be found in REFdeogen2 and in Suppl. Material.

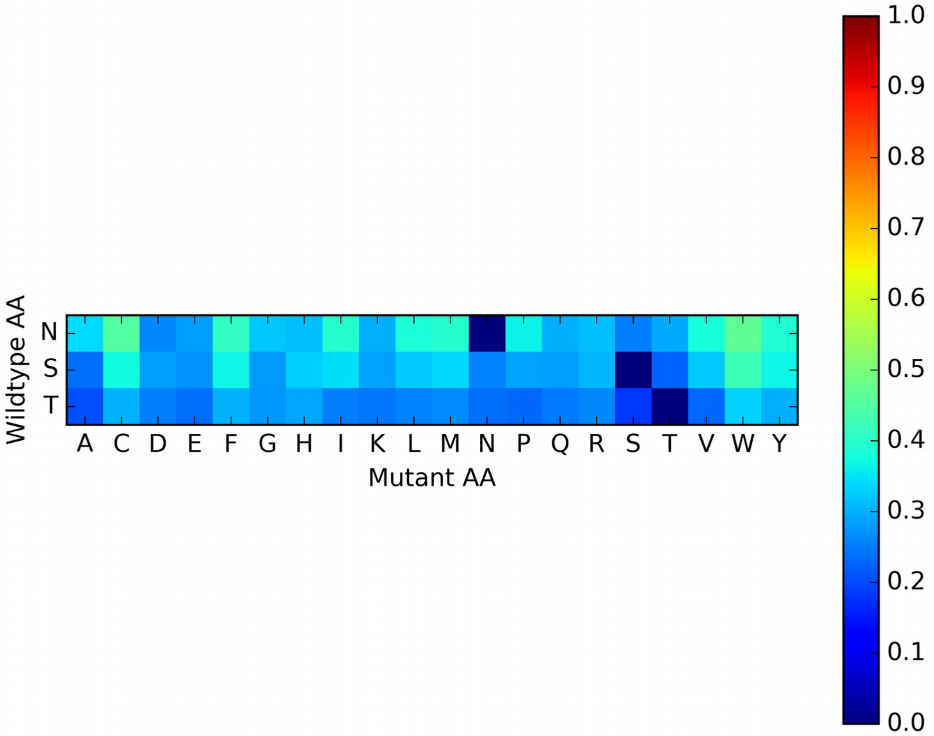

Fig. S11: Matrix showing the average deleteriousness prediction scores for variants mutating a wildtype residue (Y axis) into one of the possible 20 amino acids (X axis) when the wildtype is a glycosylated residue (see Suppl. Mat. Uniprot Annotations details).

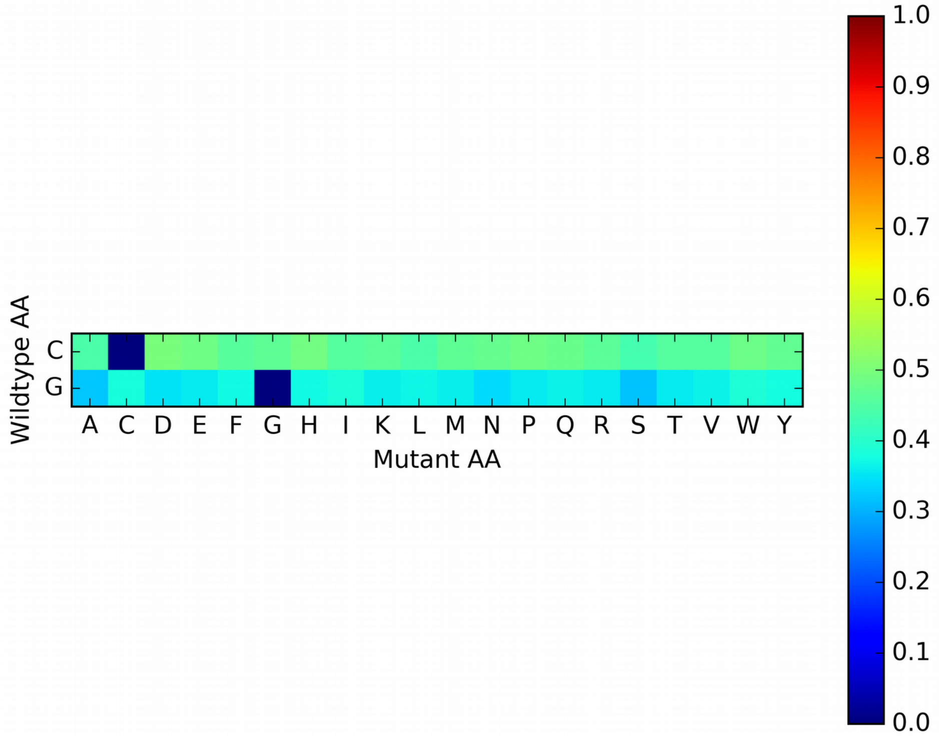

Fig. S12: Matrix showing the average deleteriousness prediction scores for variants mutating a wildtype residue (Y axis) into one of the possible 20 amino acids (X axis) when the wildtype is a lipidated residue (see Suppl. Mat. Uniprot Annotations details).

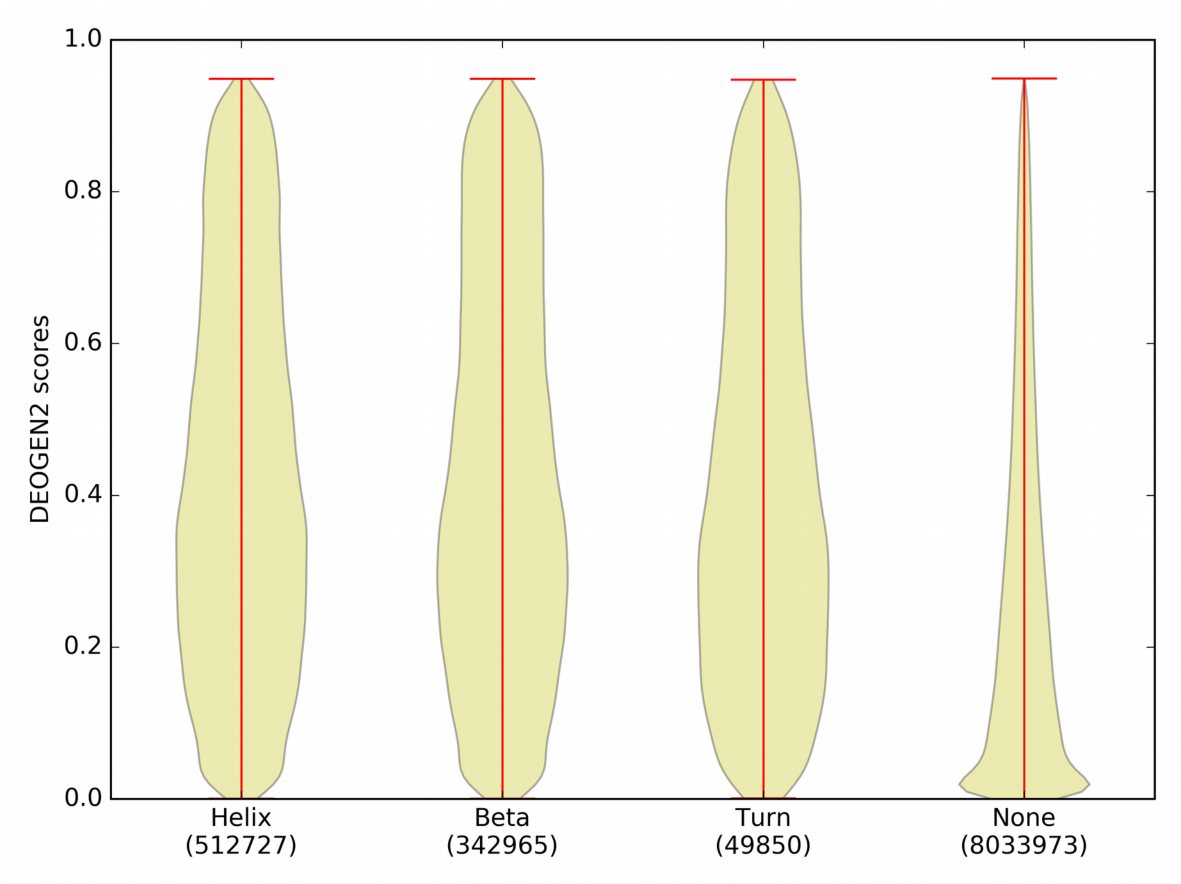

Fig. S13: Violin plots showing the distribution of the DEOGEN2 scores for different secondary structure elements in SP17.

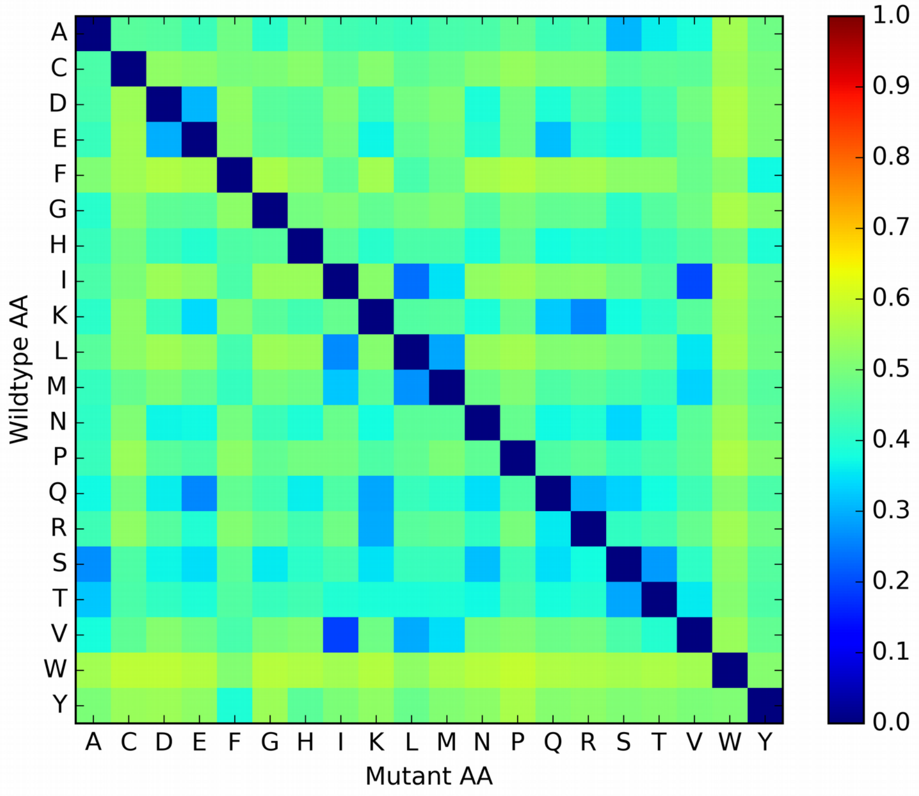

Fig. S14: Matrix showing the average deleteriousness prediction scores for variants mutating a wildtype residue (Y axis) into one of the possible 20 amino acids (X axis) when the wildtype part of an helical secondary structure element.

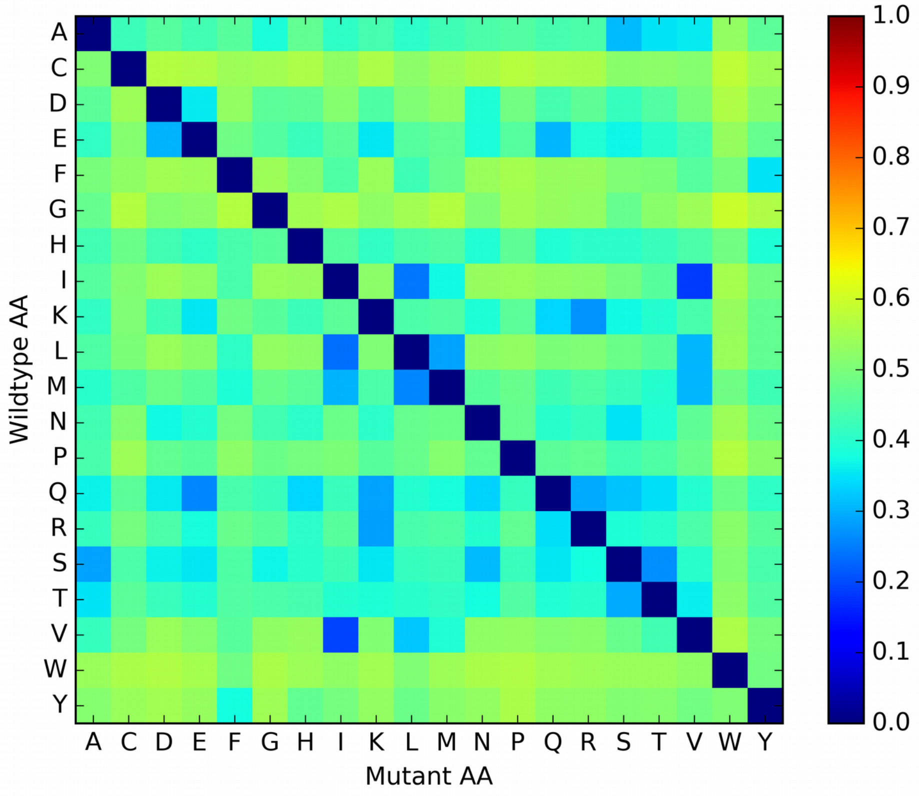

Fig. S15: Matrix showing the average deleteriousness prediction scores for variants mutating a wildtype residue (Y axis) into one of the possible 20 amino acids (X axis) when the wildtype part of an beta-sheet secondary structure element.

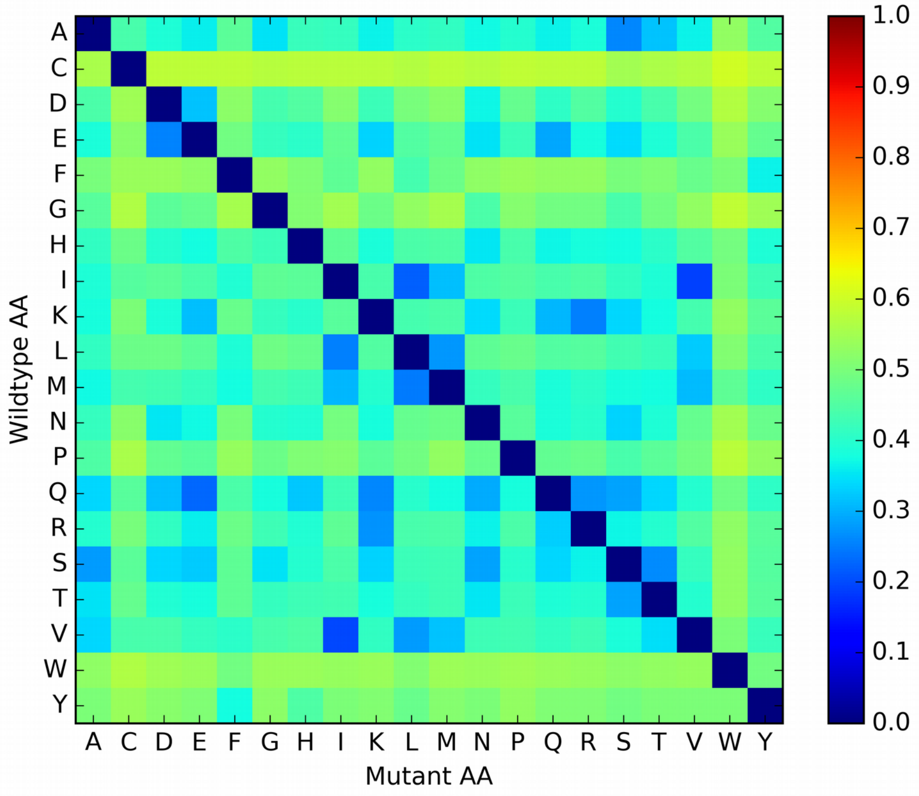

Fig. S16: Matrix showing the average deleteriousness prediction scores for variants mutating a wildtype residue (Y axis) into one of the possible 20 amino acids (X axis) when the wildtype part of an hydrogen-bonded turn secondary structure element.

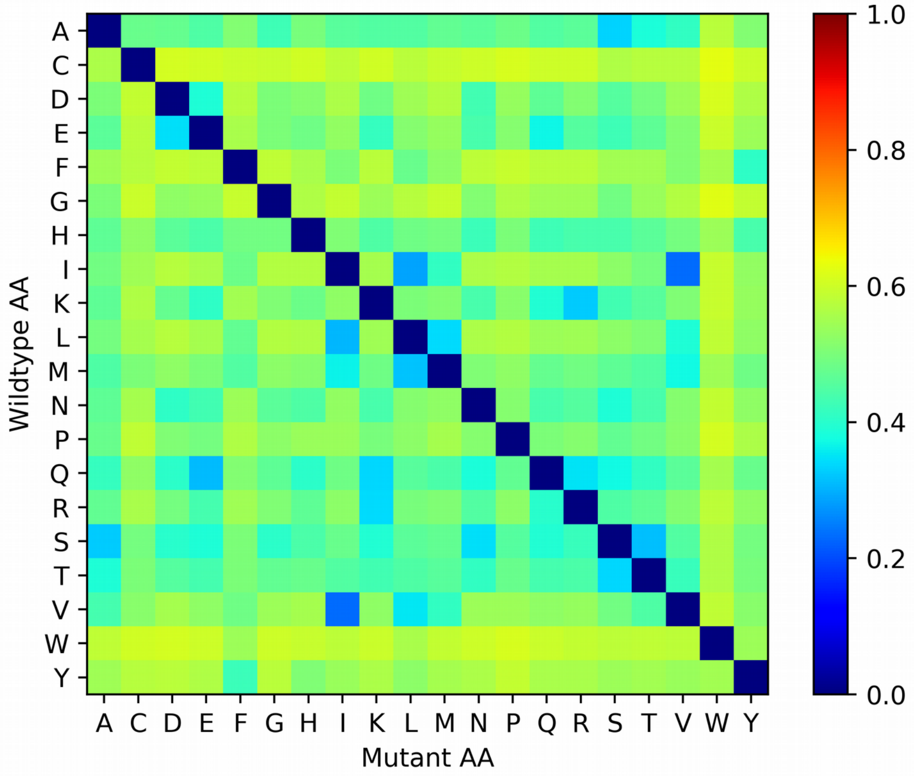

Fig. S17: Matrix showing the average deleteriousness prediction scores for variants mutating a wildtype residue (Y axis) into one of the possible 20 amino acids (X axis) when the wildtype part of an interaction patch annotated from Instruct database.

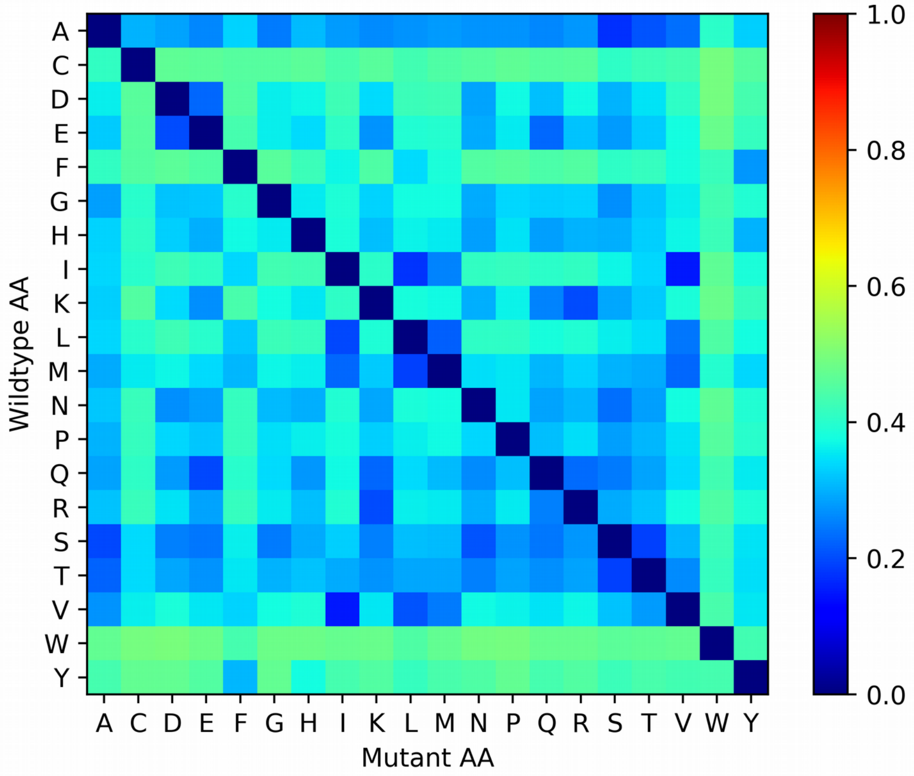

Fig. S18: Matrix showing the average deleteriousness prediction scores for variants mutating a wildtype residue (Y axis) into one of the possible 20 amino acids (X axis) when the wildtype is not part of an interaction patch annotated from Instruct database.

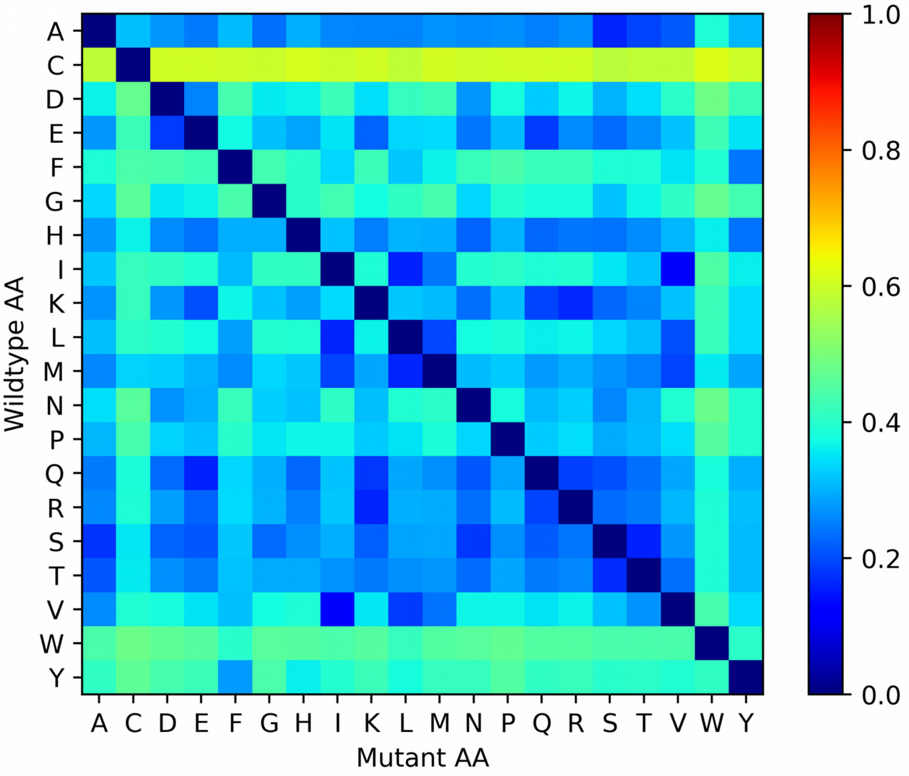

Fig. S19: Matrix showing the average deleteriousness prediction scores for variants mutating a wildtype residue (Y axis) into one of the possible 20 amino acids (X axis) when the wildtype is part of an extracellular region of a membrane protein.

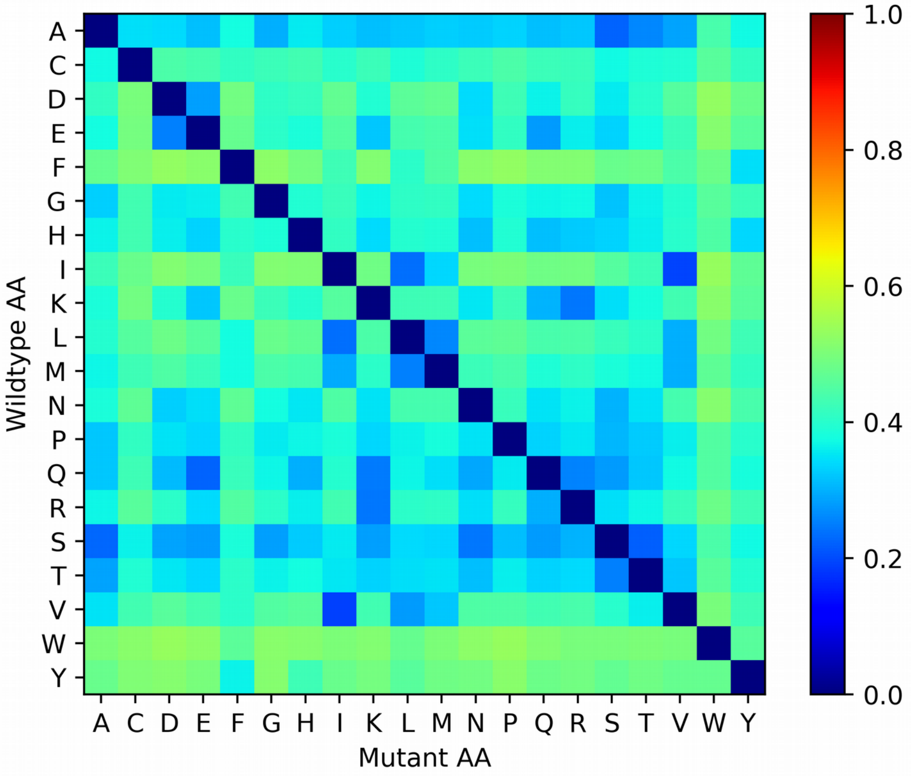

Fig. S20: Matrix showing the average deleteriousness prediction scores for variants mutating a wildtype residue (Y axis) into one of the possible 20 amino acids (X axis) when the wildtype is part of a cytoplasmic region of a membrane protein.

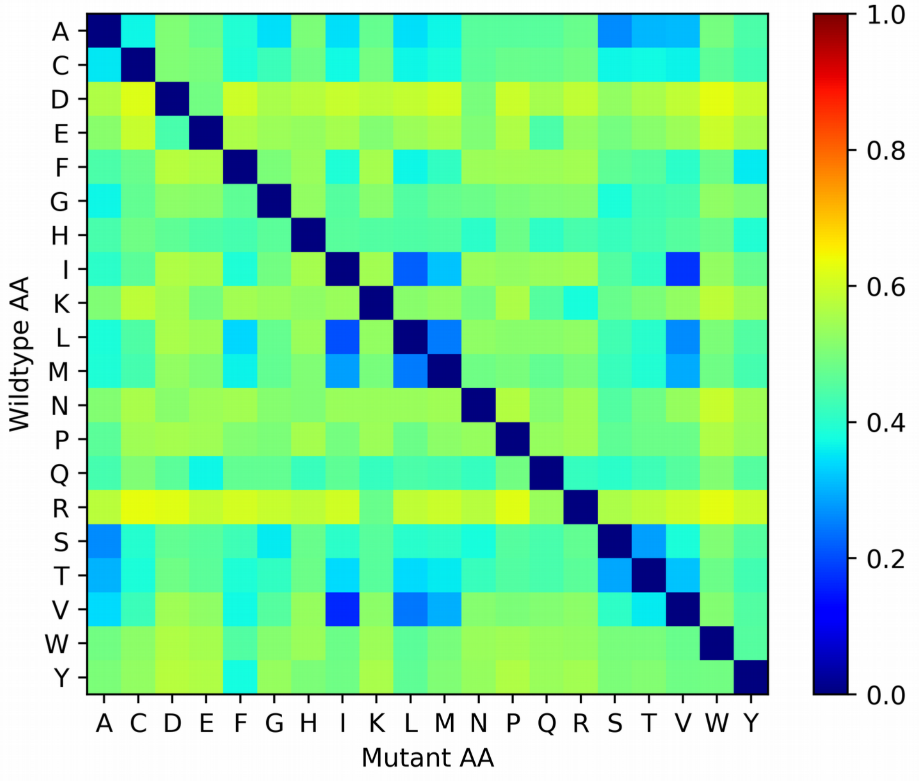

Fig. S21: Matrix showing the average deleteriousness prediction scores for variants mutating a wildtype residue (Y axis) into one of the possible 20 amino acids (X axis) when the wildtype is part of an transmembrane region of a membrane protein.

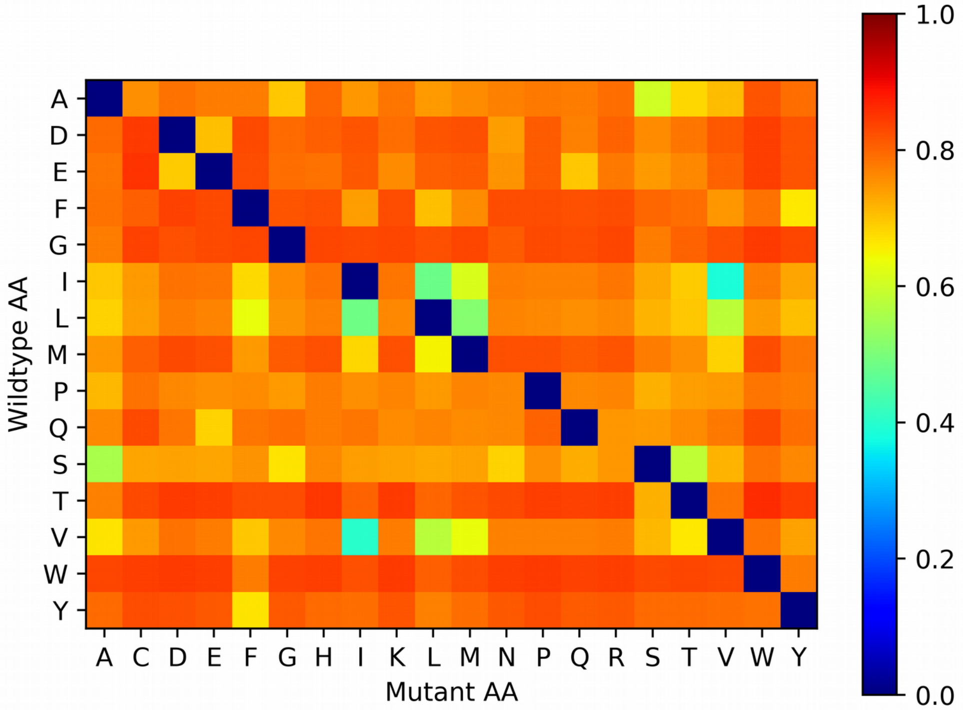

Fig. S22: Matrix showing the average deleteriousness prediction scores for variants mutating a wildtype residue (Y axis) into one of the possible 20 amino acids (X axis) when the wildtype is part of an extracellular region of a membrane protein.

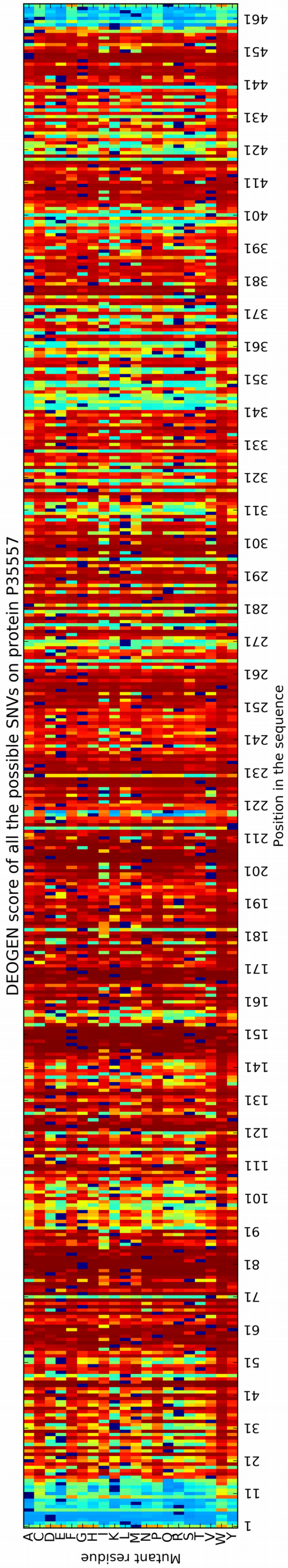

Fig. S23: Heatmap showing the complete *deleteriousness landscape* of the Human Glucokinase protein. Blue-ish scores indicate neutral variants, red-ish scores indicate deleterious predictions. The blue cells correspond to the wild-type aminoacid.

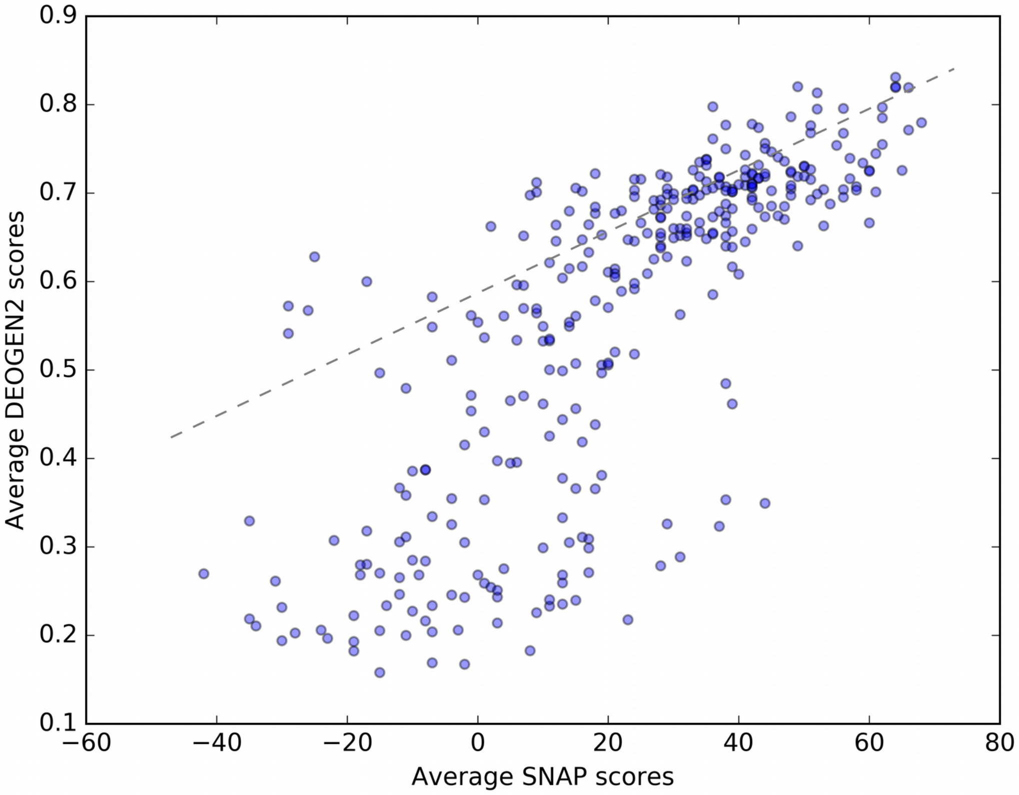

Fig. s24: Scatter plot showing the correlation between SNAP and DEOGEN2 predictions on the 159 experimentally annotated variants on the Melanocortin receptor 4 used as blind-test set.

Human Glucokinase protein (P35557) independent variants

The following text contains the list of the 24 variants on which we blind tested DEOGEN2 on P35557. The format of each row is the following:

DEOGEN2 predicted score; residue position; Wildtype → mutant ; effect of the mutation or involvement in MODY2/HHF3 diseases.

0.959065 225 I → M: Highly decreases glucokinase activity.

0.917726 434 C → F in MODY2;

0.607816 91 V → L in HHF3; increased glucokinase activity; increased affinity for glucose.

0.771272 217 D → N: Mildly increases glucokinase activity.

0.972777 191 R → W in MODY2.

0.971649 256 E → A: Inactive enzyme.

0.880906 315 L → F in MODY2;

0.596694 248 E → K: Highly decreases glucokinase activity.

0.702569 99 W → C in HHF3; increased glucokinase activity; increased affinity for glucose; increased affinity for ATP.

0.978817 68 G → D in MODY2;

0.933298 378 A → T in MODY2.

0.615586 447 R → Q in MODY2.

0.984777 152 F → L in MODY2;

0.946655 441 S → W in MODY2; decreased affinity for glucose. 2

0.90115 177 E → K: Small change in activity.

0.941721 188 A → V in MODY2.

0.924634 202 M → R in MODY2;

0.663921 65 T → I in HHF3; decreased glucokinase activity; increased affinity for glucose; unchanged affinity for ATP.

0.969764 231 N → H in MODY2;

0.868476 43 R → H in MODY2;

0.819254 442 E → K in HHF3; increased affinity for glucose. 2

0.909992 414 K → A: Small change in activity.

0.916139 223 G → S in MODY2.

0.946551 129 C → Y in MODY2.
